# An engineered glioblastoma model yields macrophage-secreted drivers of invasion

**DOI:** 10.1172/jci.insight.181903

**Published:** 2025-08-22

**Authors:** Erin A. Akins, Dana Wilkins, Zaki Abou-Mrad, Kelsey Hopland, Robert C. Osorio, Kenny K.H. Yu, Manish K. Aghi, Sanjay Kumar

**Affiliations:** 1 University of California, Berkeley–UCSF Graduate Program in Bioengineering, Berkeley, California, USA.; 2 Department of Bioengineering, University of California, Berkeley, Berkeley, California, USA.; 3 Department of Neurosurgery, Memorial Sloan Kettering Cancer Center, New York City, New York, USA.; 4 Department of Neurosurgery, UCSF, San Francisco, California, USA.; 5 Department of Chemical and Biomolecular Engineering, University of California, Berkeley, Berkeley, California, USA.

**Keywords:** Immunology, Oncology, Brain cancer, Extracellular matrix, Macrophages

## Abstract

While the accumulation of tumor-associated macrophages (TAMs) in glioblastoma (GBM) has been well documented, targeting TAMs has thus far yielded limited clinical success in slowing GBM progression due, in part, to an incomplete understanding of TAM function. Using an engineered 3D hydrogel–based model of the brain tumor microenvironment (TME), we show that M2-polarized macrophages stimulate transcriptional and phenotypic changes in GBM stem cells (GSCs) closely associated with the highly aggressive and invasive mesenchymal subtype. By combining proteomics with GBM patient single-cell transcriptomics, we identify multiple TAM-secreted proteins with putative proinvasive functions and validate TGF-β induced (TGFBI, also known as BIGH3) as a targetable TAM-secreted tumorigenic factor. Our work highlights the utility of coupling multiomics analyses with engineered TME models to investigate TAM–cancer cell crosstalk and offers insights into TAM function to guide TAM-targeting therapies.

## Abstract 2

Multi-omics analysis of 3D cell culture and patient biopsies identifies candidate glioblastoma therapeutic targets among M2 macrophage-secreted factors.

## Graphical abstract

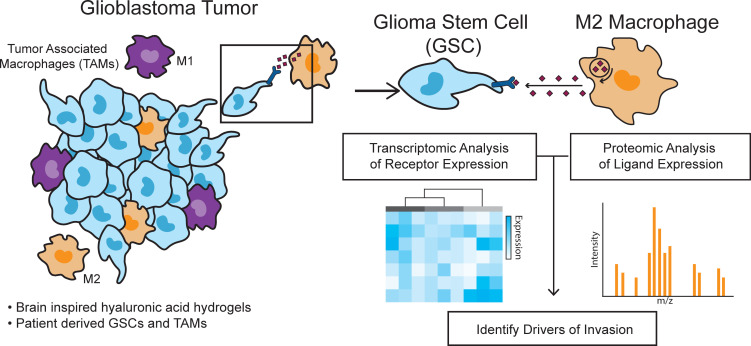

## Introduction

Glioblastoma (GBM) is the most common, lethal, and aggressive form of primary brain cancer, with a medium survival time of approximately 15 months (1). The highly invasive nature of GBM complicates surgical resection and promotes recurrence, yet it has been challenging to target invasion therapeutically due to an incomplete understanding of the underlying cellular and molecular mechanisms. Cell invasion is profoundly influenced by interactions between cancer cells and the tumor microenvironment (TME), which consists of non-neoplastic cells as well as extracellular matrix (ECM) (2). Immune cells have emerged as pivotal TME players in tumor development, making them highly attractive targets for therapeutic intervention (3, 4). GBMs exhibit a unique immune landscape dominated by tumor-associated macrophages (TAMs), which are composed of multiple subpopulations, including bone marrow–derived (BMD) macrophages and brain-resident microglia (5, 6). TAMs can comprise up to 40% of the total cells in gliomas, and their accumulation is highest in patients with the most aggressive GBM subtype (6–9). TAMs are proposed to actively regulate multiple cellular properties relevant to tumor progression, including stemness, proliferation, survival, and migration. Investigation into the mechanisms regulating these complex TAM-GBM interactions is ongoing. Several TAM-secreted factors, including TGF-β family members, interleukins, and C-C chemokine ligands, activate downstream oncogenic signaling pathways, including β-catenin signaling, AKT, NF-κB, and JAK/STAT3 (10–13). The clinical impact of TAM-tumor interactions, however, is strongly influenced by patient-specific characteristics of the tumor (location and stage) as well as macrophage cell phenotypes (14–19).

Macrophage phenotype and function are defined by a polarization state, which has traditionally been described using the M1/M2 polarization model (20, 21). TAMs are often associated with the antiinflammatory M2-like polarization state due to their high expression of antiinflammatory cytokines, scavenger receptors, proangiogenetic factors, and proteins involved in ECM organization and remodeling (22). The proinflammatory M1-like state is more commonly associated with antitumor functions such as antigen presentation and costimulation. Although the M1/M2 classification system has proven to be valuable for in vitro studies, TAM polarization is likely more nuanced than the binary M1/M2 classification would suggest, and human GBM TAMs can display both M1 and M2 gene signatures (23–26). Thus, M1 and M2 TAMs are more accurately described as antitumor (M1-like) and protumor (M2-like) macrophages. We nonetheless use the M1/M2 nomenclature here for the sake of conciseness and consistency with the literature.

An important and ongoing barrier to understanding TAM regulation of GBM invasion is the lack of in vitro platforms capturing key geometric and mechanical aspects of the GBM TME. Advances in biomaterials and tissue engineering have facilitated the development of 3D TME models (27, 28). Hydrogel-based systems are frequently used as matrices for 3D cell culture, and among these systems, hyaluronic acid (HA) is a valuable biomaterial for modeling the brain TME (29). We and others have used HA-based hydrogel platforms to identify and mechanistically dissect key pathways driving GBM invasion, metabolism, and therapeutic resistance (30–36). Despite the recent expansion of 3D brain TME models, integration of macrophages and other immune cells remains limited (37, 38).

Here we utilize a 3D HA-based hydrogel platform to test the hypothesis that TAMs establish a proinvasive microenvironment that facilitates GBM invasion. We first investigate the influence of macrophage polarization state on GBM spheroid invasion across a panel of patient-derived GBM cells. We find that factors secreted by M2-polarized macrophages induce GBM invasion. Using a microscale invasion platform that enables regional dissection and characterization of invasive cells, we identify macrophage-induced transcriptional changes in invasive GBM cells. We then characterize the macrophage secretome across polarization states using mass spectrometry, perform a GBM-TAM interaction analysis to identify receptor-ligand pairs driving invasion, and prioritize putative proinvasive TAM-secreted ligands using single-cell RNA sequencing (scRNA-seq) datasets of human GBM TAMs. We identify 2 TAM-derived secreted factors, BIGH3 (also known as TGF-β induced or TGFBI) and S100A9, that stimulate GBM invasion. We demonstrate that targeting BIGH3 and downstream mammalian target of rapamycin (mTOR) signaling reduces invasion. Overall, our study highlights the utility of engineered 3D platforms for investigating TAM–cancer cell interactions and uncovering molecular targets that may guide the development of future therapeutic intervention.

## Results

### Establishment of a brain-inspired hydrogel platform to study TAM-GBM interactions.

**Figure 1:**
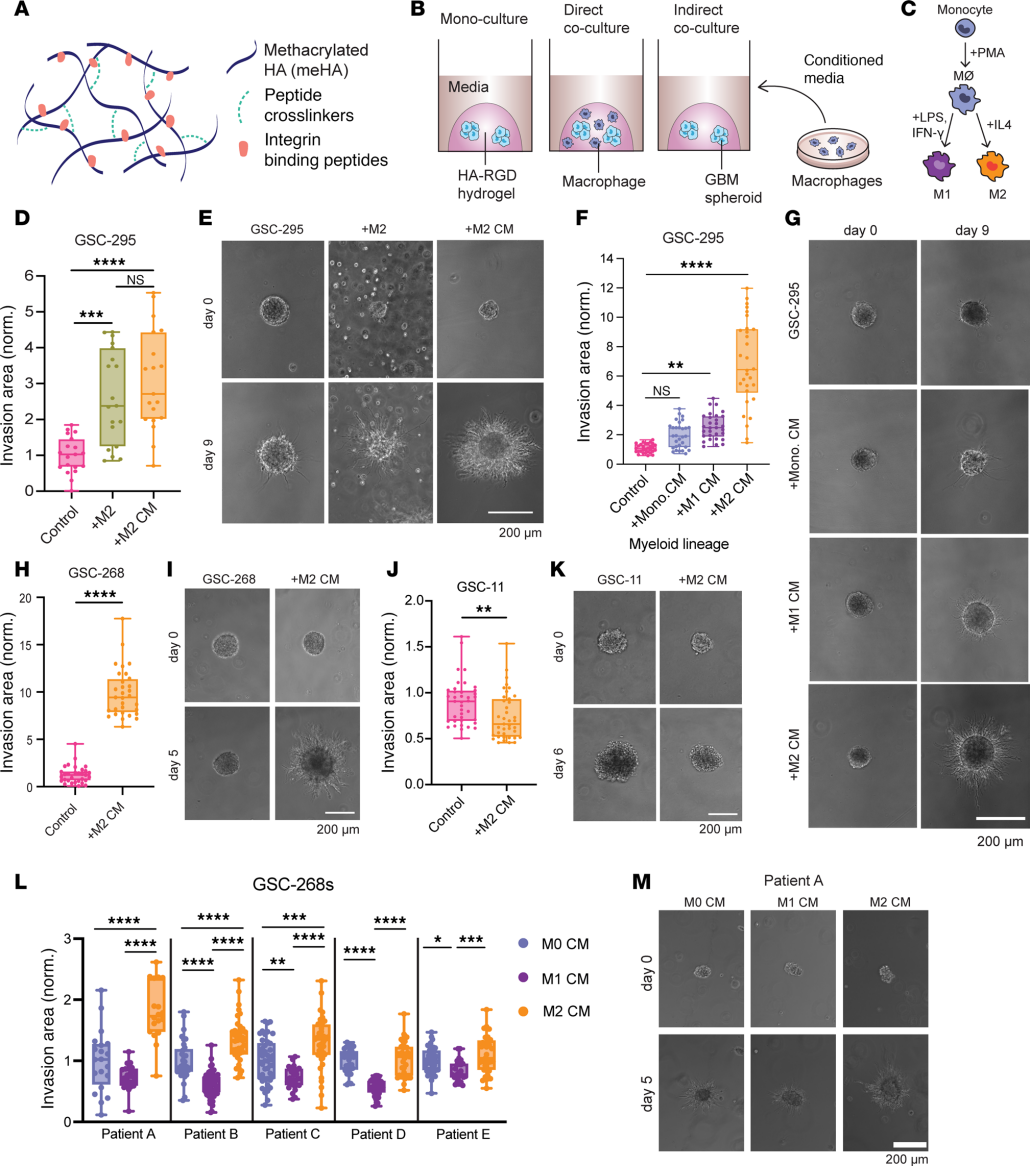
An engineered biomaterials platform to enable investigation of GBM-macrophage crosstalk. (**A**) Schematic of HA-based hydrogel. (**B**) Schematic of mono- or coculture platforms to study GBM invasion. (**C**) Schematic of THP-1–derived monocyte-to-macrophage differentiation and M1/M2 polarization protocol. (**D** and **E**) GSC-295 invasion assay with direct M2 macrophage coculture and M2 macrophage–conditioned media (CM) (*n* = 19 spheres) showing (**D**) quantification and (**E**) representative phase images. (**F** and **G**) GSC-295 invasion assay with CM from monocytes (mono.), M1 macrophages, and M2 macrophages (*n* = 30 spheres) showing (**F**) quantification and (**G**) representative phase images. (**H** and **I**) GSC-268 invasion assay with M2 CM (*n* = 31 spheres) showing (**H**) quantification and (**I**) representative phase images. (**J** and **K**) GSC-11 invasion assay with M2 CM (*n* = 39 spheres) showing (**J**) quantification and (**K**) representative phase images. (**L** and **M**) GSC-268 invasion assays with GBM patient–derived macrophage CM (*n* = 18-59 spheres) showing (**L**) quantification and (**M**) representative phase images for Patient A. Spheroid invasion results were pooled across at least 2–3 independent replicates. Statistical significance was analyzed using 1-way ANOVA followed by Tukey’s multiple-comparison test (**D**, **F**, and **L**) or an unpaired, 2-sided Student’s *t* test (**H** and **J**). *P < 0.05; **P < 0.01; ***P < 0.001; ****P < 0.0001. Scale bars: 200 μm.

We engineered a 3D in vitro brain tumor model leveraging our previously published hydrogel consisting of HA conjugated to the integrin-binding peptide GCGYGRGDSPG (HA-RGD) (39) (Figure 1A and Supplemental Figure 1A; supplemental material available online with this article; https://doi.org/10.1172/jci.insight.181903DS1). To study the influence of macrophages on GBM invasion, we performed coculture tumorsphere invasion assays by encapsulating patient-derived GBM stem cells (GSCs) in HA-RGD hydrogels with THP-1–derived M2-polarized macrophages distributed throughout the hydrogel (Figure 1, B and C, and Supplemental Figure 1B). Macrophage polarization state was confirmed via quantitative PCR (qPCR) and flow cytometry (Supplemental Figure 1, C–G). As monocultures, GSC-295 spheroids were not invasive; however, adding M2-polarized macrophages resulted in a proinvasive GSC phenotype characterized by long, thin protrusions, quantified by invasive cell area. To test whether M2 macrophages influence invasion through direct or paracrine mechanisms, we performed indirect coculture assays using M2-polarized macrophage–conditioned media (M2 CM). M2 CM treatment recapitulated the invasive phenotype of direct coculture, suggesting GSC invasion did not require macrophage-GSC physical contact (Figure 1, D and E, and Supplemental Figure 2A).

To examine the effect of polarization state on macrophage-GSC interactions, we treated GSC-295 spheroids with CM collected from monocytes and M1-polarized macrophages. GSC-295 spheroids cultured in monocyte CM (mono. CM) and M1 macrophage CM (M1 CM) were less invasive than GSC-295 spheroids cultured in M2 CM (Figure 1, F and G). These results suggest that in our system, M2 macrophages are the predominant source of proinvasive factors.

### GBM-macrophage interaction varies across a panel of patient-derived GBMs.

To determine whether M2 CM stimulated invasion of additional patient-derived GSCs, we performed tumorsphere invasion assays using a previously characterized panel of patient-derived GSCs (40). Although the influence of M2 CM on spheroids appeared to be cell line specific, the majority of GSC lines (5 out of 6) were highly invasive when treated with M2 CM (Figure 1, H and I, and Supplemental Figure 2, B–G). Interestingly, GSC-11 spheroids decreased in area when treated with M2 CM (Figure 1, J and K). We confirmed the polarization-specific response and found that M2 CM drives GSC-268 invasion greater than monocyte CM or M1 CM (Supplemental Figure 2, H and I).

To explore the clinical relevance of these findings, we obtained blood-derived macrophages from multiple patients with GBM (*n* = 5), polarized the macrophages to M1 (with LPS and IFN-γ) or M2 (with IL-4), and collected CM for GSC-268 tumorsphere invasion assays. Similarly to THP-1–derived macrophages, patient-derived macrophage CM produced a polarization-specific response in GSC invasion, with M2 CM driving a more invasive phenotype relative to M1 CM (Figure 1, L and M).

To test whether M2 CM stimulates invasion in patient-derived cells that have not been enriched for GSCs, we performed tumorsphere invasion assays with 2 GBM patient–derived xenograft lines (GBM-43 and GBM-6) (41). Interestingly, GBM-43 spheroids decreased invasion when cultured with M2-polarized macrophages or in M2 CM (Supplemental Figure 2, J and K). A similar trend was seen in GBM-6 spheroids, although it was not statistically significant (Supplemental Figure 2, L and M).

Using GSC-268 spheroids and varied concentrations of M2 CM, we found that M2 CM stimulates invasion in a dose-dependent fashion (Supplemental Figure 2N). To test whether the increased invasion was related to proliferation, we quantified the percentage of KI-67^+^ cells. There was no difference in the percentage of KI-67^+^ cells between GSCs cultured in control media and M2 CM (Supplemental Figure 2, O and P). To further investigate the influence of M2 CM on phenotypes beyond invasion, we tested whether M2 CM impacted stemness. Immunostaining showed decreased expression of SOX2, a common stemness marker, in M2 CM–treated GSC 268s compared with controls (Supplemental Figure 2, Q and R). We performed a sphere formation assay and found reduced sphere size and circularity in M2 CM–treated GSC-268s (Supplemental Figure 2, S–U).

### M2-polarized macrophage–secreted factors shift GSC transcriptional profiles toward invasion- and mesenchymal-associated signatures.

**Figure 2:**
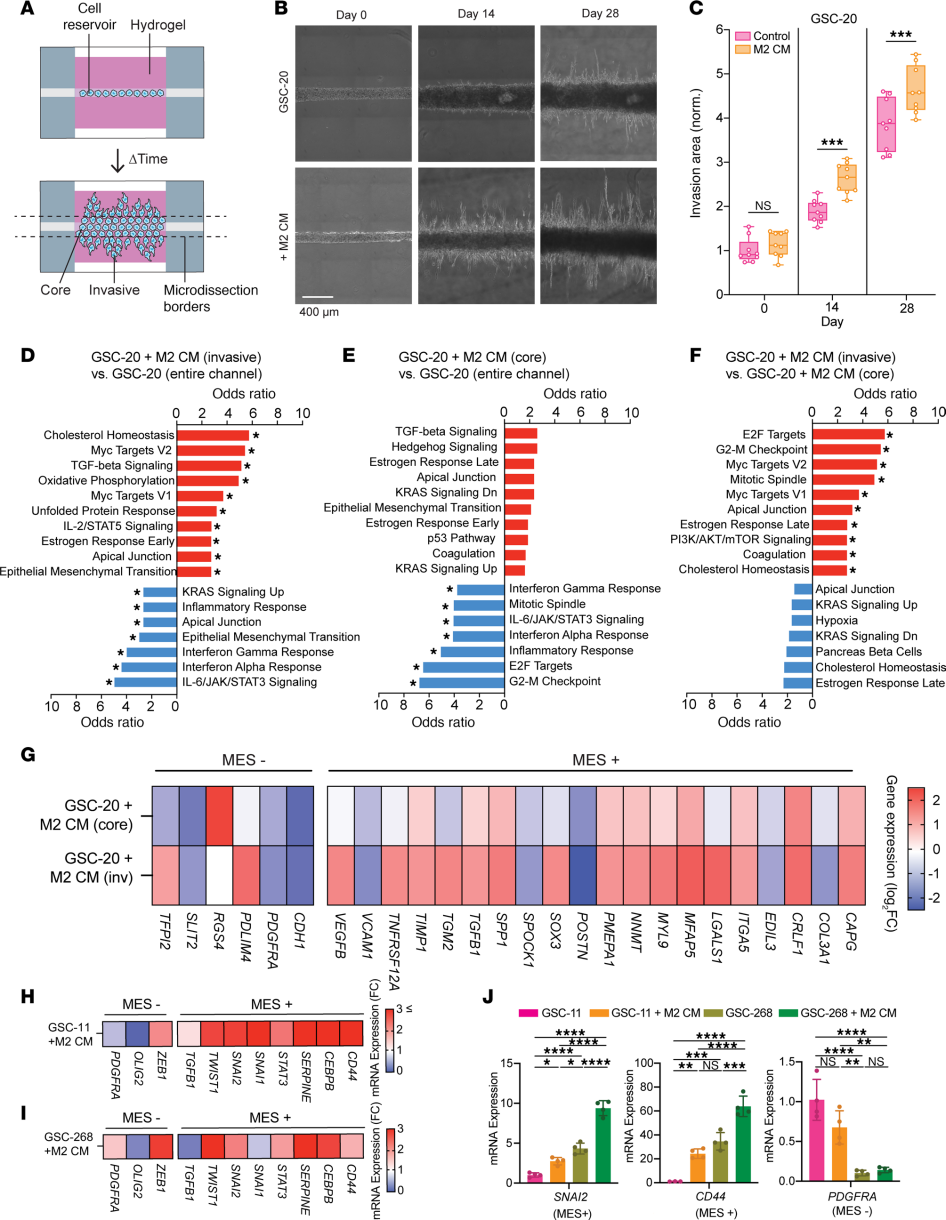
GSC transcriptional changes induced by M2 CM–mediated invasion. (**A**) Schematic of HA-based invasion device and microdissection borders (dashed lines). (**B** and **C**) GSC-20 invasion assays with M2 CM (*n* = 9 devices) showing (**B**) representative phase images and (**C**) quantification. Scale bar: 400 μm. (**D**–**F**) Cellular pathway (MSigDB) enrichment analysis of differentially expressed genes. Pathways, odds ratio, and statistical significance (*Padj < 0.05) calculated from Enrichr between (**D**) GSC-20 invasive fraction of M2 CM devices and entire channel of GSC-20 control devices, (**E**) GSC-20 core fraction of M2 CM devices and entire channel of GSC-20 control devices, and (**F**) GSC-20 invasive fraction of M2 CM devices and GSC-20 core fraction of M2 CM devices. (**G**) Heatmap showing GSC-20 relative gene expression levels of mesenchymal (MES+) and non-mesenchymal (MES–) markers obtained by bulk RNA-seq of cells isolated from invasion devices. Expression levels normalized to GSC-20 devices in control media (entire channel). (**H** and **I**) Heatmaps showing relative gene expression levels of MES+ and MES– markers obtained by qPCR of GSC-11 (**H**) and GSC-268 (**I**) cells isolated from spheroid invasion assays. Expression levels are normalized to GSCs in control media. (**J**) Relative gene expression levels of *PDGFRA*, *SNAI2*, and *CD44* across GSC lines and culture conditions (*n* = 4 biological replicates). Expression levels for each gene are normalized to GSC-11 in control media. Statistical significance was analyzed using 1-way ANOVA followed by Šídák’s multiple-comparisons test (**C**) or Tukey’s multiple-comparison test (**J**). *P < 0.05; **P < 0.01; ***P < 0.001; ****P < 0.0001.

To investigate how M2 CM alters the transcriptional profile of GSCs, we performed bulk RNA-seq on GSCs in hydrogels with or without M2 CM. We utilized our previously developed microchannel invasion platform consisting of a cylindrical cell reservoir channel embedded within a 3D hydrogel that enables the physical separation of highly invasive and noninvasive (core) cell fractions from a single device (Figure 2A) (32, 36). As expected, GSC-20s in M2 CM–treated devices were more invasive than GSC-20s in control devices (Figure 2, B and C). Following the invasion assay, we microdissected the M2 CM–treated devices to isolate the highly invasive GSCs. We performed differential gene expression analysis between cells isolated from control devices, invasive cells isolated from M2 CM–treated devices, and core cells isolated from M2 CM–treated devices. The invasive cell populations isolated from devices cultured in GSC maintenance media (control devices) did not contain enough RNA to submit for bulk RNA-seq independently and, therefore, were pooled with the core cell fractions from the same devices for transcriptomic analysis. We found invasive and core populations separated along the first principal component (PC1), indicating maximum variance along the invasive phenotype. The second principal component (PC2) generally separated GSCs based on media conditions (M2 CM vs. control media) (Supplemental Figure 3A). We identified approximately 1,000 differentially expressed genes (DEGs) between each sample comparison illustrated in the heatmap and volcano plots (Supplemental Figure 3, B–F, and Supplemental Dataset 1).

To identify pathways associated with the invasive phenotypes in response to M2 CM, DEGs were used as inputs for pathway enrichment analysis (Figure 2, D–F). M2 CM treatment led to downregulation of pathways related to Interferon Gamma Response, Interferon Alpha Response, Inflammatory Response, and IL-6/JAK/STAT3 Signaling, reflective of an immunosuppressive state marked by decreased *CXCL10*, *CXCL11*, *IFIT2*, *TLR2*, and *TLR3*. Treatment with M2 CM led to upregulation of mesenchymal (MES) pathways, including TGF-β Signaling, Apical Junction, and Epithelial-Mesenchymal Transition (EMT) (Figure 2, D and E). Invasive GSCs were associated with enrichment pathways related to Cholesterol Homeostasis, MYC Targets V1/V2, and Apical Junction (Figure 2, D and F).

Interestingly, MES pathways (TGF-β Signaling, Apical Junction, and EMT) were upregulated in both the invasive and core populations of M2 CM–treated devices, suggesting that M2 CM treatment may induce global upregulation of MES-related genes in GSCs. We further examined bulk RNA-seq expression levels of MES-associated (MES+) genes across device fractions and culture conditions (Figure 2G). We also included DEGs associated with less aggressive non-MES subtypes, designated as MES–, which have been shown to have more favorable overall patient survival compared with the MES subtype (8). Compared with GSCs isolated from control devices, GSCs cultured in M2 CM had elevated expression of MES+ genes and downregulated expression of MES– genes. The gene expression changes were seen in both M2 CM–treated core and invasive cells but were larger in the M2 CM–treated invasive cell population. Treatment with M2 CM also led to differential expression of cadherins, including the characteristic MES-associated downregulation of E-cadherin (*CDH1*) (Supplemental Figure 3G) (42).

We next explored whether M2 CM induced upregulation of MES genes across multiple patient-derived GSCs. GSC-11 spheroids, which do not invade when cultured in M2 CM, displayed an upregulation of genes associated with the MES+ phenotype (*CD44*, *CEBPB*, *SERPINE*, and *SNAI1*) and a downregulation of MES– genes (*OLIG2* and *PDGFRA*) relative to GSC-11 spheroids in control media (Figure 2H). GSC-268 spheroids, which do invade when cultured in M2 CM, also displayed an upregulation of MES+ genes (Figure 2I). To better understand the association of MES+ and MES– gene signatures with invasion, we compared gene expression levels for *PDGFRA* (MES–), *SNAI2* (MES+), and *CD44* (MES+) between GSC-11 and GSC-268. The relative expression of MES+ genes was generally lower in GSC-11 spheroids than GSC-268 spheroids regardless of media condition (Figure 2J). Additionally, GSC-11 spheroids exhibited higher gene expression levels of the MES– marker *PDGFRA* relative to GSC-268 spheroids, independent of media condition (Figure 2J).

### Identification of macrophage-derived proinvasive proteins using mass spectrometry.

**Figure 3:**
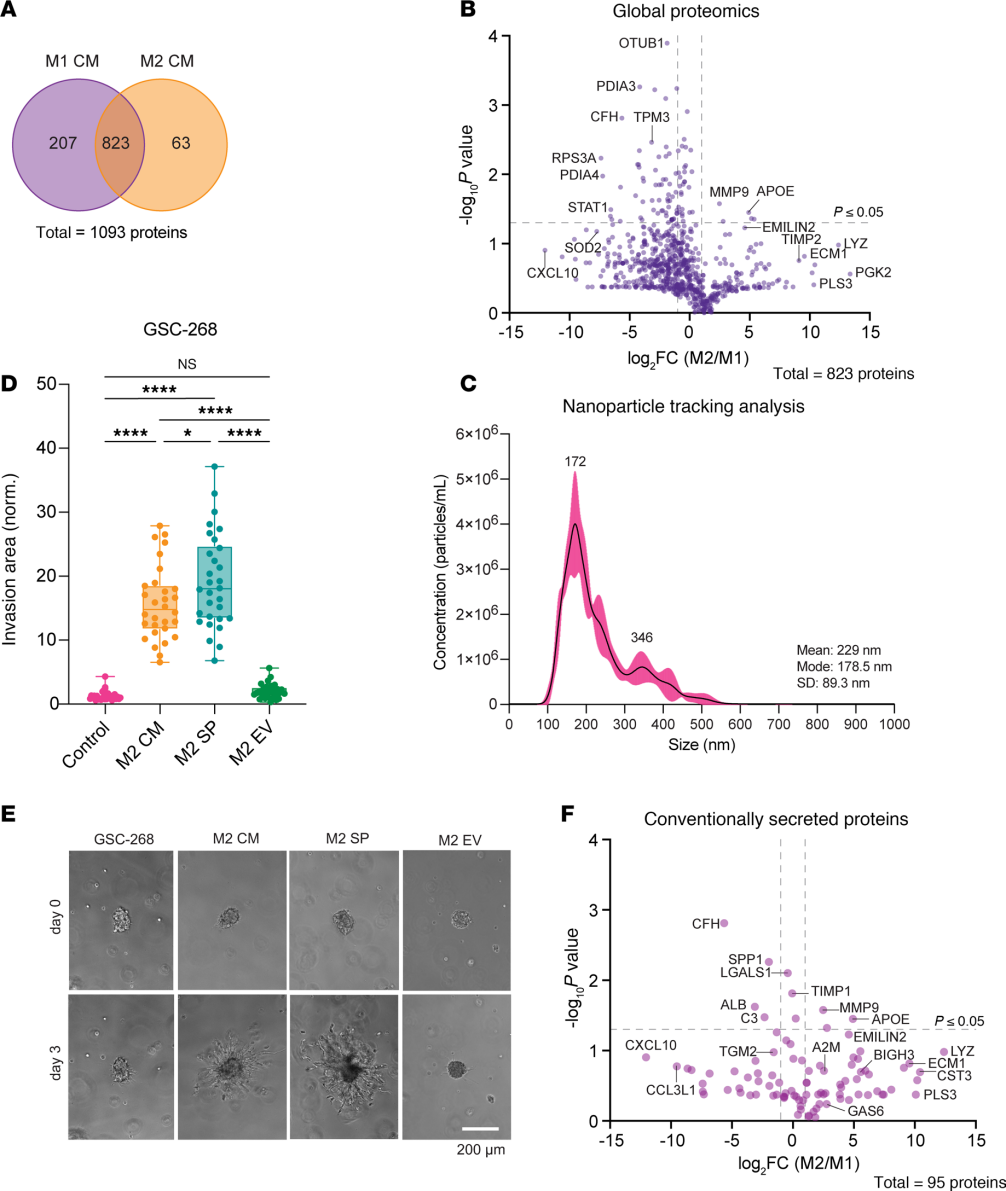
Mass spectrometry–based identification of macrophage-secreted proteins. (**A**) Venn diagram illustrating proteins identified by mass spectrometry in M1 and M2 CM. (**B**) Volcano plot of proteins identified by mass spectrometry in both M1 and M2 CM. (**C**) Nanoparticle tracking analysis (NTA) of extracellular vesicles (EVs) isolated by ultracentrifugation from M2 CM. (**D** and **E**) GSC-268 invasion assay with M2 CM, M2 soluble proteins (SP), and M2 EVs (*n* = 30 spheres) showing (**D**) quantification and (**E**) representative phase images. Scale bar: 200 μm. (**F**) Volcano plot of conventionally secreted proteins identified by mass spectrometry in both M1 and M2 CM. Spheroid invasion results were pooled across at least 2–3 independent replicates. Statistical significance was analyzed using 1-way ANOVA followed by Tukey’s multiple-comparison test (**D**). *P < 0.05; ****P < 0.0001.

To directly identify soluble factors secreted by TAMs that may induce MES+ signaling and drive GSC invasion, we performed global proteomics on macrophage CM. Using size-based exclusion filtration and heat treatment of M2 CM, we first determined that the proinvasive factor in M2 CM was greater than 10 kDa (Supplemental Figure 4A) and heat-sensitive (Supplemental Figure 4B). From these data, we hypothesized that the proinvasive factor was a protein and performed mass spectrometry to identify secreted proteins that were more abundant in M2 CM than M1 CM. Mass spectrometric analysis identified 1,093 total proteins, with 207 proteins detected only in M1 CM, 63 proteins detected only in M2 CM, and 823 proteins detected in both M1 and M2 CM (Figure 3, A and B, Supplemental Figure 4D, and Supplemental Dataset 2).

As expected, M1 CM had higher abundance of proinflammatory proteins (CXCL8, CCL3, CXCL10, CCL5, and IFI30), while M2 CM had higher abundance of antiinflammatory and ECM remodeling proteins (TIMP2, BIGH3, ECM1, ADAM10, and CSTHB) (Supplemental Figure 4E). Beyond these conventionally secreted proteins, mass spectrometric analysis identified various unconventionally secreted proteins such as OTUB1, PDIA3, and PGK2 (Figure 3B). We hypothesized that these detected proteins could have been secreted through extracellular vesicles (EVs). We isolated and purified EVs from M2 CM and performed a tumorsphere invasion assay with full M2 CM, M2 EVs, and EV-free M2 CM, referred to as M2 soluble protein fraction (M2 SP). GSC-268 spheroids invaded when treated with M2 SP and M2 CM, but were not invasive when treated with M2 EVs (Figure 3, C–E). A similar trend was observed with GSC-295 spheroids (Supplemental Figure 4, F and G). From these results, we hypothesized that the proinvasive factor was not EV bound and, therefore, computationally filtered our list of identified proteins to include only conventionally secreted proteins (Figure 3F) (43).

### GSC-macrophage interaction analysis identifies putative receptor-ligand pairs driving invasion.

**Figure 4:**
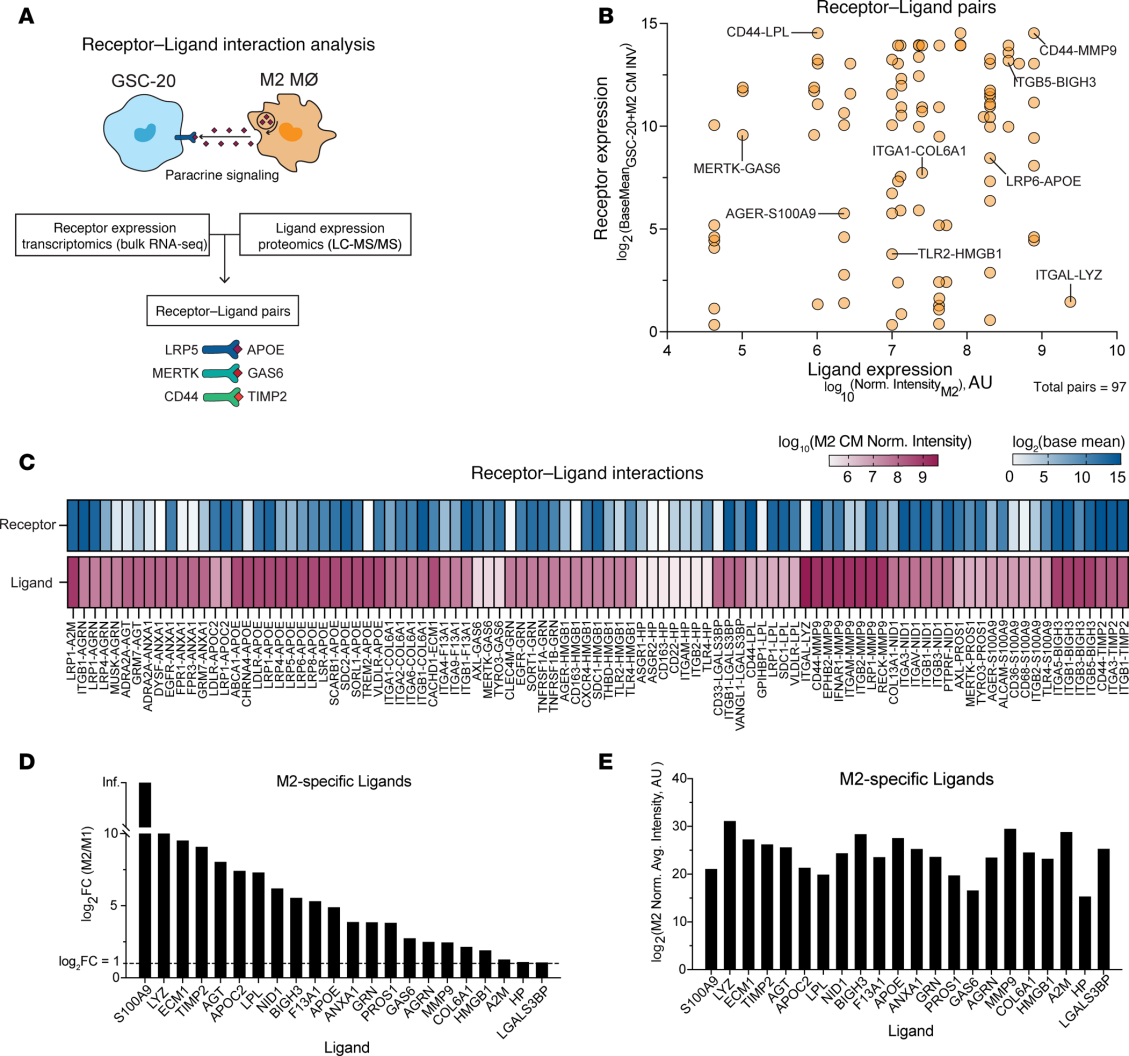
Paracrine receptor-ligand interaction analysis of GSCs and M2 macrophages. (**A**) Schematic of receptor-ligand interaction analysis workflow. (**B**) Quantification of receptor-ligand pair according to ligand expression in M2 CM (*x* axis) and receptor expression in GSCs (*y* axis). (**C**) Heatmap of receptor expression in GSCs and ligand expression in M2 CM for each receptor-ligand pair. (**D** and **E**) Bar graphs of ligands detected in M2 CM emerging from receptor-ligand analysis graphed to illustrate (**D**) average protein abundance in M2 CM compared to M1 CM and (**E**) average relative protein abundance obtained from mass spectrometry.

To identify specific receptor-ligand pairs driving invasion, we created a resource of inferred paracrine crosstalk by mapping the expression of GSC receptors to that of their cognate ligands detected in macrophage CM (Figure 4A). We identified 97 receptor-ligand pairs of which the receptor is expressed by GSC-20s and the ligand is present in M2 CM (Figure 4B and Supplemental Dataset 3). The 97 pairs included 65 unique receptors and 22 unique ligands (Figure 4C) and although all ligands were detected at higher abundance in M2 CM than M1 CM (Figure 4D), their relative expression in M2 CM varied (Figure 4E).

### Candidate M2 macrophage–secreted factors are abundant in human GBM TME and preferentially expressed by TAMs.

**Figure 5:**
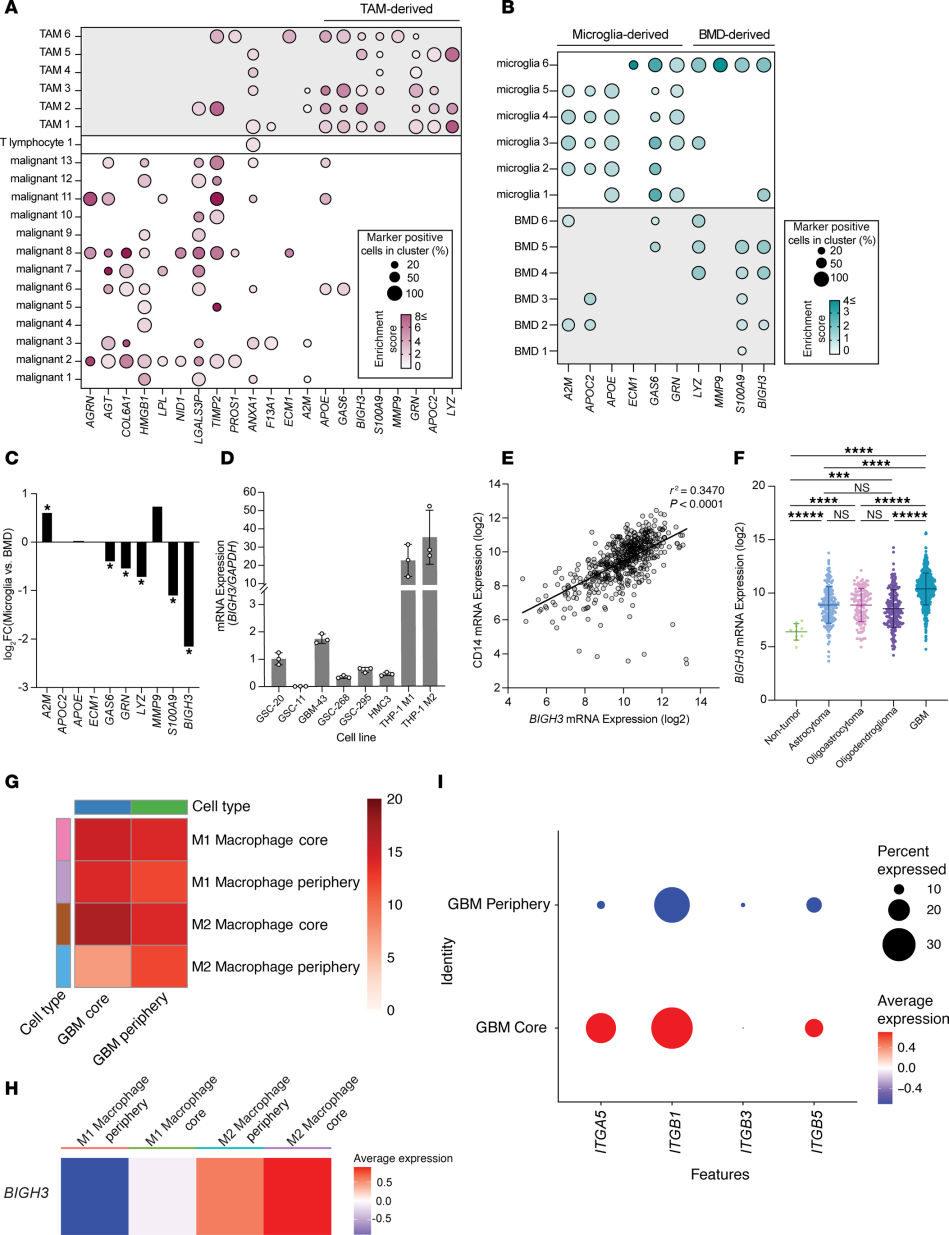
Integration of M2 macrophage–secreted ligands with published transcriptomic datasets. (**A** and **B**) Dot plots depicting gene expression profiles of M2 macrophage–secreted ligands identified by receptor-ligand analysis. Plots include (**A**) gene expression of all predicted M2 macrophage ligands across all GBM tumor cell type clusters and (**B**) gene expression of top 10 predicted M2 macrophage ligands across only GBM TAM clusters. (**C**) Differential gene expression analysis between human GBM bone marrow–derived TAMs and microglia TAMs. Genes with log_2_FC > 1 are expressed higher in microglia TAMs. (**D**) Relative BIGH3 mRNA expression obtained from qPCR across a panel of cell lines (*n* = 3 biological replicates). (**E** and **F**) Gene expression data from TCGA database queried and downloaded using GlioVis data portal for visualization and analysis of brain tumor expression datasets. (**E**) Simple linear regression plot and analysis of CD14 and BIGH3 gene expression in GBM tumors. (**F**) BIGH3 mRNA expression (log_2_) across glioma tumor stage. (**G**–**I**) Analysis of scRNA-seq dataset using CellChat. (**G**) Interactions between macrophages and GBM cells in the core and periphery. (**H**) BIGH3 expression in macrophage populations in the core and periphery. (**I**) BIGH3 receptor expression in GBM core and periphery. Statistical significance was analyzed using 1-way ANOVA followed by Tukey’s multiple-comparison test (**F**). ***P < 0.001; ****P < 0.0001; *****P < 0.00001.

We next investigated the relevance of candidate ligands to human GBM tumors using a publicly available scRNA-seq dataset (44). We focused our analysis on ligands that were predominantly expressed by TAM clusters: *ECM1*, *A2M*, *APOE*, *GAS6*, *BIGH3*, *S100A9*, *MMP9*, *GRN*, *APOC2*, and *LYZ* (Figure 5A). To predict whether these ligands were specific to microglia or BMD TAMs, we probed a subclustered version of the same dataset. Every ligand was identified in at least 1 TAM cluster but some ligands were preferentially expressed by either microglia or BMD TAMs (Figure 5B). Since most TAMs in GBM are of monocytic origin, and CM from polarized human microglia did not drive GSC invasion (Supplemental Figure 6, A and B), we prioritized ligands predominantly expressed by BMD TAMs (*BIGH3*, *S100A9*, and *LYZ*). We validated these findings using a second publicly available human GBM scRNA-seq dataset (45) and found that the top 3 DEGs upregulated in BMD TAMs were again *BIGH3*, *S100A9*, and *LYZ* (Figure 5C).

We focused further mechanistic investigation on *BIGH3* (which encodes the protein BIGH3, also known as TGFBI), as this secreted extracellular protein was one of the top ligands emerging from the multiomics receptor-ligand analysis. We measured *BIGH3* across a panel of human cells and found that M2-polarized macrophages had the highest *BIGH3* expression compared with GBM cells and microglia (Figure 5D).

To determine whether *BIGH3* expression varied by glioma grade, we used the GlioVis data portal for visualization and analysis of brain tumor expression datasets (46). In patients with GBM, we found a positive linear correlation between expression levels of *BIGH3* and the monocytic marker *CD14* (Figure 5E). Additionally, *BIGH3* expression was highest in GBM tumors compared with lower-grade gliomas and nontumor regions (Figure 5F).

To further understand regional variations in *BIGH3* in human tumors, we probed an additional human scRNA-seq dataset (26). We used the CellChat algorithm to investigate interactions between GBM cells and macrophages located in the core and periphery. M2 macrophages were found to have a greater number of interactions with GBM cells in the core when compared with the periphery (Figure 5G). Furthermore, M2 macrophages both in the core and periphery expressed *BIGH3* more highly than M1 macrophages in either location (Figure 5H). GBM cells in the core were found to express several known integrin receptors for BIGH3, including *ITGA5*, *ITGB1*, *ITGB3*, and *ITGB5* (Figure 5I).

### TAM-secreted BIGH3 promotes GSC invasion.

**Figure 6:**
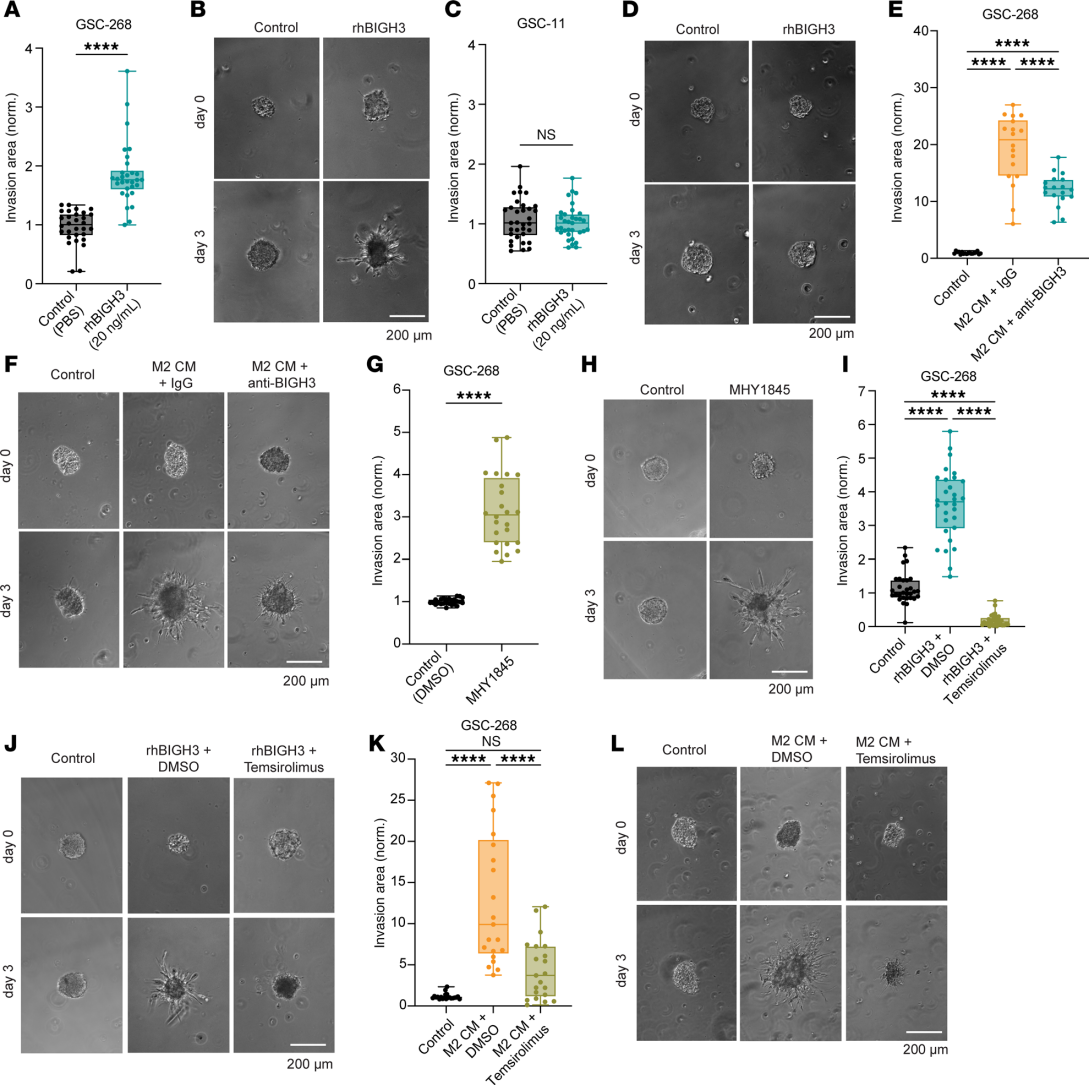
M2 macrophage–induced GSC invasion is mediated by secreted BIGH3 and is associated with active mTOR pathway. (**A** and **B**) GSC-268 invasion assay with 20 ng/mL rhBIGH3 (*n* = 30 spheres) showing (**A**) quantification and (**B**) representative phase images. (**C** and **D**) GSC-11 invasion assay with 20 ng/mL rhBIGH3 (*n* = 33 spheres) showing (**C**) quantification and (**D**) representative phase images. (**E** and **F**) GSC-268 invasion assay with M2 CM neutralized with 10 μg/mL IgG (rabbit) or 10 μg/mL anti-BIGH3 (*n* = 18 spheres) showing (**E**) quantification and (**F**) representative phase images. (**G** and **H**) GSC-268 invasion assay with 10 μM mTOR activator MHY1845 (*n* = 24 spheres) showing (**G**) quantification and (**H**) representative phase images. (**I** and **J**) GSC-268 invasion assay with 20 ng/mL rhBIGH3 and 10 μM mTOR inhibitor temsirolimus (*n* = 30 spheres) showing (**I**) quantification and (**J**) representative phase images. (**K** and **L**) GSC-268 invasion assay with M2 CM and 10 μM mTOR inhibitor temsirolimus (*n* = 21 spheres) showing (**K**) quantification and (**L**) representative phase images. Spheroid invasion results were pooled across at least 2–3 independent replicates. Statistical significance was analyzed using an unpaired, 2-sided Student’s *t* test (**A**, **C**, and **G**) or a 1-way ANOVA followed by Tukey’s multiple-comparison test (**E**, **J**, and **K**). ****P < 0.0001. Scale bars: 200 μm.

We next tested whether recombinant human BIGH3 (rhBIGH3) was sufficient to drive GSC invasion. We found that while rhBIGH3 induced GSC-268 spheroid invasion (Figure 6, A and B), rhBIGH3 interestingly had no effect on GSC-11 spheroids, which also did not invade with M2 CM (Figure 6, C and D). To test the functional contributions of BIGH3 in M2 CM–mediated invasion, we performed invasion assays using a blocking antibody targeting BIGH3 (anti-BIGH3). GSC-268 spheroids cultured in M2 CM and anti-BIGH3 were less invasive than spheroids cultured in M2 CM with IgG isotype control antibody (Figure 6, E and F).

We next explored another top hit from our multiomics analysis: S100 calcium binding protein A9 (S100A9). GSC-268 spheroids treated with recombinant human S100A9 (rhS100A9) exhibited increased invasion relative to spheroids cultured in control media and this interaction was inhibited using the small molecule S100A9 inhibitor, ABR-238907 (ABR) (Supplemental Figure 5, C and D). Interestingly, ABR did not change the invasive capacity of GSC-268 spheroids cultured in M2 CM, so although S100A9 stimulates invasion in our system, it does not appear to be a targetable proinvasive secreted factor in M2 CM (Supplemental Figure 5, E and F).

We explored the intracellular signaling pathways activated by BIGH3 that could be driving invasion. BIGH3 is a secreted ECM protein, and its expression is regulated by TGF-β signaling (47). According to our bulk RNA-seq analysis, TGF-β signaling and *TGFB1* are both upregulated in highly invasive GSCs (Supplemental Figure 5G). To test the possibility that BIGH3 and M2 CM promote invasion through TGF-β signaling, we directly induced TGF-β signaling with recombinant human TGF-β1 (rhTGF-β1). The addition of rhTGF-β1 did not change the invasive capacity of GSC-268 spheroids, suggesting that the induction of TGF-β signaling with rhTGF-β1 is not sufficient to drive GSC invasion (Supplemental Figure 5, H and I).

Another pathway upregulated in highly invasive GSCs was the P13K/AKT/mTOR signaling pathway, which is abnormally activated in tumors to promote growth, metastasis, and invasion (48). We first asked whether mTOR activation was sufficient to drive invasion and found that inducing mTOR signaling with an mTOR activator (MHY1845) induced GSC-268 spheroid invasion (Figure 6, G and H). We next tested whether BIGH3-promoted GSC invasion was mediated by mTOR signaling. Indeed, inhibiting mTOR (temsirolimus) decreased rhBIGH3-mediated GSC invasion, as GSC-268 spheroids treated with rhBIGH3 and temsirolimus were significantly less invasive than spheroids treated with rhBIGH3 and vehicle control (DMSO) (Figure 6, I and J). We then determined what portion of M2-mediated GSC invasion was driven by mTOR by testing the ability of temsirolimus to block M2 CM–mediated GSC invasion. We found that GSC-268 spheroids treated with M2 CM and temsirolimus were significantly less invasive than spheroids treated with M2 CM and vehicle control (DMSO) (Figure 6, K and L). Inhibition of mTOR signaling through temsirolimus blocked BIGH3- and M2 CM–mediated GSC invasion, suggesting that in our system, M2 CM and BIGH3 stimulate invasion through the mTOR pathway.

## Discussion

Tumor–immune cell interactions within the GBM TME represent a promising and largely untapped set of targets for limiting disease progression and improving patient outcomes. Progress in identifying these targets is limited by a lack of physiologically mimetic culture platforms that support both tumor cell invasion and incorporation of TAMs. Our 3D HA models address this need by enabling the measurement of GBM invasion in response to TAM-secreted factors. Using this model, we showed that THP-1–derived M2 CM promotes invasion across multiple patient-derived GSCs, and we saw a similar trend using CM from GBM patient-derived macrophages. Unbiased multiomics analysis of GBM and TAM populations identified the proinvasive TAM-secreted factors BIGH3 and S100A9. BIGH3 has recently been recognized as a key component of the tumor ECM, with both tumor-suppressing and tumor-promoting functions, depending on tumor type and stage (49). BIGH3 has also been proposed as a potential MES subtype signature gene based in part on its ability to promote growth and motility of continuous GBM cell lines (50, 51). Additionally, macrophage-derived BIGH3 has been shown to promote stemness and growth in GSCs (52). Similarly, S100A9 is overexpressed in various human cancers and linked to TME immunosuppression and therapeutic resistance (53–57). Our work provides additional evidence that BIGH3 and S100A9 are active drivers of GBM invasion and suggests that they are primarily derived from the BMD TAM population.

Our findings begin to deconstruct complex TAM–GBM cell interactions and support ongoing therapeutic efforts to inhibit M2 function or repolarize M2 macrophages toward M1-like polarization states (58–61). While there have been reports of high M2 TAM accumulation in MES tumors, the mechanism behind this correlation remains unclear, although it has been proposed that MES tumors attract M2 TAMs (8, 62). Our work expands on prior studies investigating the relationship between TAM abundance and GBM heterogeneity by proposing the possibility that M2 TAM–secreted factors directly induce MES transition in tumor cells. Several GSC-secreted factors, including IL-6, CCL2, VEGFA, and periostin, have been shown to recruit and polarize TAMs; however, further research is needed to determine to what extent TAM recruitment is influenced by MES classification (10). TAMs and GSCs may potentially form a bidirectional feedback loop, where M2 TAMs induce a highly invasive MES state in GSCs, leading to further M2 polarization of TAMs. This notion is further supported by our finding that high expression of MES gene signatures correlates with highly invasive behavior, a connection previously demonstrated in breast cancer but not fully elucidated in brain tumors (63–65).

This study reveals notable patient-to-patient heterogeneities in GSC-TAM interactions. For example, the majority of patient-derived GSC lines invaded when stimulated with M2 CM, except for GSC-11. While we did not investigate the reasons for this lack of response, prior characterization of GSC-11 (40) revealed several differences from GSC-20 (highly invasive with M2 CM), including greater hypermethylation and substantial enrichment of glioma–CpG island methylator phenotype (G-CIMP) signature genes (40). Positive G-CIMP status has previously been correlated with lower immune infiltration (66). GSC-11 has lower expression of CD44 than GSC-20 (40), which could explain differences in invasion given the critical role of CD44 in adhesion to and migration through HA (67). An additional example of heterogeneity is the differential response of GSC-268 invasion to patient TAM CM, which likely reflects the inherent intertumoral heterogeneity in GBM. It would be interesting to repeat these studies with GSCs and TAMs derived from the same patient.

In this study, the mTOR pathway emerged as a targetable regulator of GSC-TAM interactions and GSC invasion. Although the exact mechanisms connecting BIGH3, mTOR signaling, and MES transition remain unclear, BIGH3 has several known integrin-binding motifs (68, 69) and mTOR activation can occur through integrin-dependent mechanisms (70, 71). Given the importance of mTOR signaling in cell growth, proliferation, metabolism, and survival, this pathway is a potential therapeutic target, and our data support ongoing exploration of mTOR pathway inhibition as combination therapy for patients with GBM (48).

In addition to invasion, TAMs have been shown to support other protumor functions, including stemness and regulating immune cell activation. Bulk RNA-seq analysis showed downregulation of inflammatory response pathways in GSCs exposed to M2 CM, which may indicate TAMs are contributing to immunosuppression. Interestingly, we saw that M2 CM treatment decreased expression of the stemness marker SOX2 and led to reduced sphere area and circularity during sphere formation assays, indicating a reduction in stemness. While macrophages have promoted stemness in other systems, in this study, M2 CM appeared to support differentiation toward a MES subtype characterized by decreased cell-cell contacts resulting in smaller spheres with lower circularity and increased cell-ECM adhesion, indicated by a highly invasive phenotype in HA-based matrices.

By exploiting engineered 3D TME models to investigate functional contributions of the TAM secretome on GBM invasion, we show that the effects of TAM secretome on GBM are context specific and vary by macrophage ontogeny and polarization state, as well as GBM stemness and mesenchymal characterization. BIGH3 and other TAM-secreted factors merit further mechanistic and therapeutic study, including the roles these factors may play in priming the TME for invasion. It is also important to elucidate specific TAM subsets or polarization states beyond the traditional M1/M2 model responsible for the secretion of proinvasive factors, as well as identify the TME features driving the presence of these key TAM polarization states and subtypes. Regional variations in TAM-GBM interactions will also be important to understand given the microenvironmental differences between the tumor core and edge. Finally, understanding how TAM infiltration and function relate to GBM transcriptional heterogeneity and tumor recurrence would focus efforts to target TAMs in patients who would most benefit.

## Methods

### Sex as a biological variable.

Cells used in this study were sourced from both male and female patients. Patient sex of all cell lines used is summarized in Supplemental Table 1.

### Cell lines.

GBM-43 and GBM-6 (Mayo Clinic) were cultured in Dulbecco’s modified Eagle medium (DMEM) (Thermo Fisher Scientific) supplemented with 10% (vol/vol) FBS (Corning, MT 35-010-CV), 1% (vol/vol) penicillin-streptomycin (Thermo Fisher Scientific), and 1% (vol/vol) GlutaMax (Thermo Fisher Scientific, 35-050-061). Bulk GBM cell lines (GBM-43, GBM-6) were harvested using 0.25% Trypsin-EDTA (Thermo Fisher Scientific, 25200-072) and passaged less than 30 times.

GSC-11, GSC-268, GSC-28, GSC-262, GSC-20, and GSC-295 lines were provided by the University of Texas M.D. Anderson Department of Neurosurgery. The GSC lines were propagated as neurospheres in DMEM/F12 50:50 1× (Corning, 10-090-CV) supplemented with 2% (vol/vol) B-27 supplement (Gibco), 20 ng/mL EGF (R&D Systems, 236-EG), and 20 ng/mL FGF (R&D Systems, 233-FB). All GSCs were harvested using Accutase cell detachment solution (Innovative Cell Technologies, 490007-741) and passaged less than 20 times.

THP-1 cells (ATTC, TIB-202) were cultured in RPMI-1640 medium (Sigma-Aldrich, R8758-500ML) supplemented with 10% (vol/vol) FBS (Corning, MT 35-010-CV), 1% (vol/vol) penicillin-streptomycin (Gibco, 15140122), 1% (vol/vol) MEM nonessential amino acids (Thermo Fisher Scientific, 11140050), 1% (vol/vol) sodium pyruvate (Gibco, 11360070), 1% (vol/vol) GlutaMax (Thermo Fisher Scientific, 35-050-061), 1% (vol/vol) HEPES (1 M, Gibco, 15630080), and 0.05 mM 2-mercaptoethanol (Sigma-Aldrich, M3148-25ML). THP-1 cells were cultured at 3 × 10^5^ cells/mL, split when they reached 8 × 10^5^ cells/mL, and passaged less than 30 times.

THP-1 cells were differentiated and polarized following previously reported protocols (72). In brief, cells were differentiated at a concentration of 5 × 10^5^ cells/mL with 100 nM phorbol 12-myristate 13-acetate (PMA; PeproTech, 1652981) in complete RPMI-1640 media for 24 hours followed by 3 days of resting. Macrophages were polarized to M1 with 50 ng/mL human IFN-γ (Bio Basic, RC217-17) and 50 ng/mL LPS (Sigma-Aldrich, L2630-10MG) for 48 hours. Macrophages were polarized to M2 with 50 ng/mL IL-4 (Bio Basic, RC212-15-5) for 48 hours (Supplemental Figure 1B). M1/M2 polarization was confirmed by measuring mRNA expression of established M1 and M2 markers with qPCR (Supplemental Figure 1, C and D), noting morphological changes associated with differentiation and polarization (Supplemental Figure 1E), and measuring surface M1/M2 marker expression via flow cytometry (Supplemental Figure 1F).

Patient peripheral blood mononuclear cells (PBMCs) were collected from multiple patients with GBM (*n* = 5) at Memorial Sloan Kettering Cancer Center (New York) and isolated via magnetic-activated cell sorting for CD14^+^ cells. CD14^+^ cells were plated in RPMI-1640 plus 10% FBS for 24 hours before differentiating with 50 ng/mL M-CSF for 48 hours. The postdifferentiation and polarization protocol mirrored that of the THP-1s. Briefly, the differentiated macrophages were left to rest for 24 hours before treating with polarization stimulus for 48 hours (M1: 50 ng/mL LPS and 50 ng/mL IFN-γ; M2: 50 ng/mL IL-4). Polarization of macrophages was confirmed via qPCR of M1 (*TNFA* and *IL1B*) and M2 (*CD209*) markers and imaging of morphological changes (Supplemental Figure 1, H and I).

HMC3 cells (ATCC, CRL-3304) were cultured in DMEM/F12 50:50 1× (Corning, 10-090-CV) supplemented with 10% (vol/vol) FBS (Corning, MT 35-010-CV) and 1% (vol/vol) penicillin-streptomycin (Thermo Fisher Scientific). Microglia were polarized following the same THP-1 protocol described above. Cells were seeded at a density of 3 × 10^4^ cells/cm^2^, split when confluence reached 90%, and passaged less than 20 times.

All cells were screened for mycoplasma and validated by short tandem repeat (STR) analysis every 6 months at the University of California Cell Culture Facility. For experiments in hydrogels, 0.5% penicillin and streptomycin (Gibco, 15140122) and 0.125 μg/mL Amphotericin B solution (Sigma-Aldrich, A2942) was added to cell culture media. Additional information on GBM source and classification can be found in Supplemental Table 1.

### Me-HA synthesis.

HA hydrogels were synthesized as previously described (39). Briefly, methacrylic anhydride (Sigma-Aldrich, 94%, 760-93-0) was used to functionalize sodium hyaluronate (Lifecore Biomedical, Research Grade, 66–99 kDa, HA60K-5) with methacrylate groups. The extent of methacrylation per disaccharide was quantified by ^1^H-NMR spectroscopy and found to be approximately 85% for materials used in this study. To add integrin-adhesive functionality, Me-HA was conjugated via Michael addition with the cysteine-containing RGD peptide Ac-GCGYGRGDSPG-NH_2_ (Anaspec, AS-62349) at a concentration of 0.5 mmol/L.

### Hydrogel rheological characterization.

Hydrogel stiffness was characterized by shear rheology via a Physica MCR 301 rheometer (Anton Paar) with an 8-mm parallel plate geometry for g = 0.5% and f = 1 Hz. Frequency was controlled to be between 50 and 1 Hz for the frequency sweep at a constant strain (g = 0.5%), and the modulus saturation curve with time was obtained under oscillation with constant strain (g = 0.5%) and frequency (f = 1 Hz). The temperature of the gel solution was controlled (T = 37°C) with a Peltier element (Anton Paar) and the sample remained humidified throughout the experiment.

### HA hydrogel crosslinking.

To form hydrogels, 6% (w/w) Me-HA conjugated with 0.5 mM integrin-binding peptide (RGD) was crosslinked with a protease-cleavable peptide (KKCG-GPQGIWGQ-GCKK, Genscript) in phenol red–free and serum-free DMEM (Thermo Fisher Scientific, 21-063-029) containing GBM cell spheroids. Unless otherwise stated, all experiments in this study utilized a final 1.5% (w/w) Me-HA hydrogel crosslinked with 3.064 mM peptide to yield a shear modulus of approximately 200 Pa, which is within the range of values typically reported for brain tissue (73–75).

### Generation of spheroids.

Tumorspheres were fabricated using Aggrewell 400 24-well plates (Stemcell Technologies, 34415). Briefly, 1.2 × 10^5^ cells were seeded into a single well of the Aggrewell plate to form spheroids consisting of 100 cells after a 48-hour incubation at 37°C and 5% CO_2_.

### 3D spheroid invasion assay.

Spheroids were resuspended in phenol red–free and serum-free DMEM at a density of 1.5 spheroids/μL and used as solvent for HA hydrogel crosslinking. For direct coculture experiments, THP-1–derived macrophages were added to DMEM for a final concentration of 3,500 cells/μL hydrogel. For fluorescently labeled coculture experiments, THP-1s were fluorescently labeled after differentiation and polarization with CellTracker dye (Thermo Fisher Scientific, C2925) following the manufacturer’s protocol prior to encapsulation with GSC spheroids.

### CM collection.

To prepare CM, THP-1s were cultured in DMEM/F12 50:50 1× (Corning, 10-090-CV) at a density of 1 × 10^6^ cells/mL. For PBMC-derived-macrophage CM, cells were cultured in Macrophage-SFM media (Gibco, 12065074). After 48 hours, CM was collected and centrifuged at 130*g* for 5 minutes to remove dead cells and debris and passed through a 25 mm, 0.45 μm sterile cellulose acetate filter (VWR, 76479-040). CM was used immediately or stored at –20°C prior to use. For indirect coculture experiments, cell culture supplements (B-27 or FBS) were added to CM immediately before use. Multiple freeze/thaw cycles were avoided. For invasion assays with patient macrophage CM, invasive area was normalized to spheroids treated with CM from unpolarized macrophages (control) to determine whether the observed differences in GSC invasion were directly due to macrophage polarization states rather than heterogeneity among patient samples.

### Heat treatment of CM.

CM was harvested as described above. Samples were placed in a dry heat bath at 100°C. Samples were incubated for 1 hour and cooled to room temperature before use. If applicable, cell culture supplements (B-27 or FBS) were added to CM immediately before use.

### EV purification.

We used ultracentrifugation and nanoparticle tracking analysis to isolate and characterize M2-polarized macrophage–derived EVs. CM was harvested as described above. Microvesicles were removed by centrifugation at 5,000*g* for 15 minutes. Supernatant was collected and ultracentrifuged at 140,000*g* (28,000 rpm) for 1.5 hours in an SW-28 rotor (Beckman Coulter). The supernatant was collected (soluble protein fraction) and the crude EV pellet was resuspended in 1× PBS and ultracentrifuged at 160,000*g* for 2 hours. The supernatant was discarded, and the crude EV pellet was resuspended in 0.02-μm-filtered PBS.

### Nanoparticle tracking analysis.

EV sizes and quantities were estimated using the NanoSight NS300 instrument equipped with a 405-nm laser (Malvern Instruments), analyzed in the scatter mode. Silica 100-nm microspheres (Polysciences) served as a control to check instrument performance. Vesicles collected as described above were diluted 1,000-fold with 0.02-μm-filtered PBS. The samples were introduced into the chamber automatically, at a constant flow rate of 50 for 5 repeats of 60-second captures at camera level 13 in scatter mode with NanoSight NTA 3.1 software (Malvern Instruments). The size was estimated at detection threshold 5 using NanoSight NTA 3.1, after which “experiment summary” and “particle data” were exported. Particle numbers in each size category were calculated from the particle data, in which “true” particles with track length greater than 3 were pooled, binned, and counted with Excel (Microsoft). M2 CM contained EVs at a concentration of 1.67 × 10^8^ ± 7.47 × 10^6^ particles/mL with 100–400 nm diameters (mean: 229 ± 89.3 nm, mode: 178.5 nm) according to nanoparticle tracking analysis of concentrated EVs (Figure 3C). For the invasion assay, EVs were reconstituted in GSC maintenance media at a concentration of 1.67 × 10^8^ particles/mL, the mean concentration of EVs in M2 CM.

### Invasion device fabrication.

Invasion devices were fabricated following a modified version of our previously published protocol (32). In brief, an acrylic mold was made by laser cutting and stacking 1.5-mm-thick acrylic posts and acrylic slide (CLAREX Precision Thin Sheet, 1.5 mm, Astra Products) on top of a glass microscope slide (Fisherbrand, 12-550-A3). Next, 0.00695 mm outer diameter cleaning wires (Hamilton, 18302) were inserted into the double channel posts to serve as wire and syringe guides during hydrogel casting and cell seeding. Polydimethylsiloxane (PDMS) was fabricated by mixing a 10:1 mass to mass ratio of Sylgard 184 elastomer with the initiator (Dow Corning) and the mixture was pipetted into the acrylic mold and wire assembly and cured at 80°C for 2 hours. After curing, the wires, acrylic slide, and acrylic posts were carefully removed. A razor blade with an acrylic guide was used to cut a 5 mm by 75 mm rectangle in the center of the PDMS and the acrylic laser cut lid was attached to the PDMS and glass slide with epoxy. New 0.00695 mm outer diameter cleaning wires were reinserted into the PDMS guides and the entire device was UV treated for 10 minutes and stored in a cold room prior to use. Laser cutter designs (Adobe Illustrator files) are available in Supplemental Figure 6.

On the day of experiment, devices were brought to room temperature and the HA hydrogel solution was cast within the acrylic molds around the wire and incubated for 1 hour in a humidified 37°C chamber. After crosslinking, devices with hydrogels and wire were submerged in cell culture media for at least 10 minutes, before removal of the wire which left an open channel. Afterwards, 100,000 single cells were seeded into each open channel and the wires were reinserted into the device to plug each end of the cell reservoir. The entire device was placed into a 4-well plate and bathed in 10 mL of medium. Medium was changed every 3 days.

### Invasion quantification.

For invasion analysis in tumorsphere invasion assays, spheroids were imaged every 2 days using a Nikon Eclipse TE2000 microscope with a Plan Fluor Ph1 ×10 objective. Images were acquired using NIS-Elements Software. For each spheroid, total spheroid area was outlined in ImageJ (NIH) and normalized to total day 0 spheroid area. For experiments with direct coculture of GBM and THP-1 cells, GSCs were transduced with CAG-GFP lentivirus (Cellomics, PLV-10057-50) and selected with 1 μg/mL puromycin (Invitrogen, A1113803) prior to experiments.

For quantification of invasion in devices, cells in devices were imaged every 7 days using a Nikon Eclipse TE2000 microscope with a Plan Fluor Ph1 ×10 objective. Images were acquired and stitched using NIS-Elements Software. For each device, total cell area was outlined in ImageJ and normalized to total day 0 cell area.

### RNA extraction.

For RNA extraction of cells on tissue culture plastic, phenol-free total RNA was extracted from cells directly in cell culture wells using an RNeasy Plus Micro Kit with gDNA eliminator columns (Qiagen, 74034) following the manufacturer’s protocol. For RNA extraction of cells in invasion devices, devices were carefully disassembled, and if applicable, the invasive cells were physically separated from the noninvasive “core” cells based on distance from channel using a scalpel. The invasive cell populations isolated from devices cultured in GSC maintenance media were not large enough to submit for bulk RNA-seq independently and were pooled with the core cell fractions from the same devices. For RNA extraction of tumorsphere invasion assays, hydrogels and cells were placed in Eppendorf tubes. All samples were treated with 10,000 U/mL hyaluronidase from bovine testes, type IV-S (Sigma-Aldrich, H3884) for 15 minutes with agitation until the hydrogel was fully degraded. Afterwards, RNA was extracted using TRIzol Reagent (Invitrogen, 15596018) according to the manufacturer’s recommendations. In brief, 1 × 10^6^ cells were mixed with 100 μL TRIzol Reagent and the solution was vortexed for 30 seconds. Chloroform (20 μL; Sigma-Aldrich, c2432) was added to the mixture and the solution was placed on ice for 15 minutes. Samples were centrifuged at 18,000*g* for 10 minutes and the clear RNA layer was carefully transferred to a new Eppendorf tube. Each RNA sample was mixed with a 1:1 volume of 2-propanol (Sigma-Aldrich, 190764-4L) and 0.5 μL glycogen, RNA grade (Thermo Fisher Scientific, R0551). After an overnight incubation at –20°C, samples were centrifuged at 18,000*g* for 10 minutes at 4°C and the RNA pellet was rinsed twice by aspirating the supernatant, adding 400 μL 75% ethanol, and centrifuging at 18,000*g* for 1 minute. Following the second rinse, the supernatant was carefully and completely removed, and RNA pellets were incubated at room temperature until visibly dry. RNA was dissolved in 30 μL RNase-free DNase-free distilled H_2_O and RNA concentration and purity were measured with a Nanodrop.

### cDNA synthesis.

cDNA was synthesized from extracted RNA using an iScript cDNA Synthesis Kit (Bio-Rad, 1708891) following the manufacturer’s protocol.

### RNA-seq and differential gene expression analysis.

Isolated RNA was sent to Novogene Corporation Inc. for library construction, quality control, and sequencing. In brief, mRNA was purified from total RNA using poly-T oligo–attached magnetic beads. After fragmentation, the first-strand cDNA was synthesized using random hexamer primers followed by the second-strand cDNA synthesis. The library was ready after end repair, A-tailing, adapter ligation, size selection, amplification, and purification. The library was checked with a Qubit and real-time PCR for quantification and bioanalyzer for size distribution detection. Quantified libraries were be pooled and sequenced on Illumina platforms, according to effective library concentration and data amount. Data were filtered to remove low-quality reads and sequences containing adapters and DEGs were identified using DESeq2 (https://github.com/thelovelab/DESeq2). Statistically significant DEGs were chosen based on fold change (FC) using a statistical cutoff of |log_2_FC| of 1 or greater and adjusted *P* value of less than 0.05.

### Pathway enrichment analysis.

We used Enrichr (76–78) to perform enrichment analysis of gene lists using the Molecular Signatures Database (MSigDB) (79) hallmark gene set collection. MSigDB Pathways were ranked by odds ratio.

### Receptor-ligand interaction analysis.

To infer receptor-ligand interactions between GSCs and M1/M2 THP-1 macrophages, we compared our RNA-seq data of GSC-20s and our global proteomics data of M1/M2 macrophage CM to a database of 3,631 receptor-ligand interactions (80, 81), which was downloaded and analyzed in RStudio (RStudio, Inc., 2022.12.0+353; https://dailies.rstudio.com/version/2022.12.0+353/). We first filtered the M1/M2 macrophage CM dataset to identify conventionally secreted proteins using The Human Protein Atlas database of predicted secreted proteins (43). We then annotated cognate pairs coexpressed by GSC-20s for which the base mean transcript expression of the receptor in the GSC-20 + M2 CM (invasive fraction) sample is 1 or greater. This produced 255 receptor-ligand pairs for which the receptor is expressed by GSC-20s and the ligand is expressed by M1/M2 macrophages. To identify the receptor-ligand interactions dependent on macrophage polarization state, we grouped our inferred receptor-ligand pairs into M2- and M1-specific interactions using FC = (M2 normalized intensity)/(M1 normalized intensity). FC ≥ 2 indicated M2-specific interactions, 2 < FC > 0.5 indicated pan-macrophage interactions, and FC ≤ 0.5 indicated M1-specific interactions. This analysis yielded 100 M1-specific interactions, 97 M2-specific interactions, and 58 pan-macrophage interactions (Supplemental Dataset 3).

### Acquisition and analysis of scRNA-seq datasets.

DEGs from scRNA-seq analysis of 9 patient GBMs were downloaded from Cui et al. (44), who had obtained 10× Genomics scRNA-seq data from the NCBI’s Gene Expression Omnibus (GEO) database under GSE131928 (82). The sequencing analyses were conducted on samples from 9 fresh GBM tumors from adult patients in Massachusetts General Hospital. Dimensionality reduction, unsupervised clustering, and identification of cell types and marker genes in different clusters were performed as previously described (44). In brief, single-cell transcriptomes for a total of 16,201 cells were retained after initial quality controls and were differentiated into 22 different clusters, which were further sorted into 3 main cell types by comparing their gene expression characteristics using the following marker genes: malignant cells (*GAP43*, *GPM6B*, *SEC61G*, and *PTN*), TAMs (*C1QA*, *C1QB*, *C1QC*, *TYROBP*, and *CD68*), and T lymphocytes (*CD3G*, *GZMH*, *IL2RB*, *PR1F*, and *ICOS*). Pct.1 represents the percentage of cells where the gene is detected in specific cluster and pct.2 represents the percentage of cells on average in all the other clusters where the gene is detected. Enrichment score was calculated by dividing pct.1 by pct.2. Clusters 2, 3, 6, 7, 9, and 19 represent TAMs, cluster 17 represents T lymphocytes, and clusters 0, 1, 4, 5, 8, 10–16, 18, 20, and 21 represent malignant cells. To comprehensively characterize the signatures of TAMs in GBM, the cells in the macrophage and microglia groups were subclustered and reanalyzed. In total, 5,896 cells were sorted and hierarchically sorted into 14 clusters, as described in ref. (44). Clusters 4–7, 10, and 11 represent resident microglia TAMs and clusters 0–3, 8, 9 and 13 represent BMD TAMs.

DEGs from human blood–derived and microglial TAMs were downloaded from published data by Müller et al., who performed scRNA-seq on a cohort of 19 total patients comprising 5,455 TAMs (DESeq-adjusted *P* < 1 × 10^–3^) (45). The scRNA-seq datasets were mined for genes encoding proteins that were detected by mass spectrometry in M2 CM and identified in our GSC-macrophage interaction analysis. Data were downloaded and analyzed in RStudio.

To investigate regional differences in BIGH3 expression and GBM receptor expression, we utilized the scRNA-seq dataset from Darmanis et al. (26). The R package Seurat was used to further define the immune subpopulations and isolate M1 and M2 macrophages using previously defined canonical macrophage markers as well as markers that were suggestive of M1 and M2 subtypes (83). Once these cells were defined from the preexisting immune cluster, this updated RNA-seq dataset was used to create a CellChat object, which was analyzed using the standard CellChat analysis workflow (84). Briefly, the expression of ligand and receptor markers was used to elucidate communication between cell clusters to construct a signaling network. The analysis was all conducted using R version 4.2.2, Seurat 5.0.3, and CellChat 1.6.1.

### Acquisition of The Cancer Genome Atlas gene expression.

The Cancer Genome Atlas (TCGA) data were queried and downloaded using the GlioVis (46) data portal for visualization and analysis of brain tumor expression datasets. Data were graphed and analyzed in GraphPad Prism.

### qPCR.

qPCR was carried out using Applied Biosystems PowerUp SYBR Green Master Mix (Thermo Fisher Scientific, A25918) and primers following the manufacturer’s recommended protocol in a Bio-Rad CFX Connect Real-Time PCR cycler with a 5 μM final forward and reverse primer concentration. Ct values were calculated using CFX Maestro software accompanying the real-time cycler and relative gene expression was calculated using the 2^–ΔΔCt^ method. Each gene expression was internally normalized by the expression level of housekeeping gene run on the same qPCR batch. Primer sequences were designed using either Primer-BLAST (85) or PrimerBank (86). Primer sequences are listed in Supplemental Table 2.

### Flow cytometry.

THP-1s were differentiated and polarized as described above at 1 × 10^6^ cells/well in 6-well plates. Cells were lifted with a cell scraper, centrifuged at 400*g* for 4 minutes, and washed with PBS. Cells were incubated with Live/Dead Zombie Aqua Dye (BioLegend, 77143) and Fc blocker (Human TruStain FcX, BioLegend, 422301) for 15 minutes on ice before centrifuging and washing with PBS. Cells were then stained for M1 and M2 markers for 45 minutes on ice before 2 additional PBS washes. Finally, cells were resuspended in 500 μL of FACS buffer (1% BSA, 0.1% sodium azide, in 1× PBS), transferred to flow tubes, and analyzed by flow cytometry with a FACSymphony A3 (BD Biosciences). To prepare compensation controls, 1 drop of beads (~50 μL) was mixed with 0.5 μL of antibody and incubated for 15 minutes before adding 450 μL of PBS. Compensation and flow data analysis was performed in FlowJo v10 (FlowJo). Antibodies and flow reagents are listed in Supplemental Table 3.

### Immunostaining.

GSC268s were seeded in HA hydrogels either as single cells or spheroids, depending on the experiments. At the end of the experiment, gels were washed with PBS and fixed with 4% paraformaldehyde in PBS for 30 minutes. Next gels were washed with PBS and then permeabilized and blocked with 0.3% Triton X-100 and 5% goat serum in PBS for 30 minutes. Gels were incubated with the primary antibody in 1% goat serum in PBS for 72 hours on a shaker at 4°C. The gels were then washed with PBS and then incubated with secondary antibodies and DAPI for an additional 24 hours at 4°C. Hydrogels were then imaged using confocal microscopy (Zeiss LSM 880).

### Sphere formation assay.

GSC268s were seeded at 1 × 10^5^ cells/mL as single cells in 24-well plates in either control media or M2 CM media. After 3 days, the wells were imaged. Sphere area and circularity were measured using ImageJ.

### Mass spectrometry sample preparation.

To prepare CM for mass spectrometric analysis, M1- and M2-polarized macrophages were cultured in DMEM/F12 50:50 1× (Corning, 10-090-CV) at a density of 1 × 10^6^ cells/mL. After 48 hours, CM from M1- and M2-polarized macrophages was collected and centrifuged at 130*g* for 5 minutes to remove dead cells and debris. The CM was then added to Amicon Ultra-4 Centrifugal Filter Unit tubes (Millipore, UFC8010-24) and concentrated by centrifugation at 10,000*g* for 15 minutes, resulting in a 20-fold reduction in volume (V_i_ = 4.0 mL; V_f_ = 0.2 mL).

In preparation for mass spectrometric analysis, samples underwent a trypsin in-solution protein digest followed by sample desalting. Peptides were analyzed in biological triplicate on a Thermo Fisher Scientific Orbitrap Fusion Lumos Tribid mass spectrometry system equipped with an Easy nLC 1200 ultrahigh-pressure liquid chromatography system interfaced via a Nanospray Flex nanoelectrospray source.

Samples were injected on a C18 reverse phase column (25 cm × 75 μm packed with ReprosilPur C18 AQ 1.9 μm particles). Peptides were separated by an organic gradient from 5% to 30% acetonitrile in 0.02% heptafluorobutyric acid over 180 minutes at a flow rate of 300 nL/min. Spectra were continuously acquired in a data-dependent manner throughout the gradient, acquiring a full scan in the Orbitrap (at 120,000 resolution with an AGC target of 400,000 and a maximum injection time of 50 ms) followed by as many MS/MS scans as could be acquired on the most abundant ions in 3 seconds in the dual linear ion trap (rapid scan type with an intensity threshold of 5000, HCD collision energy of 32%, AGC target of 10,000, maximum injection time of 30 ms, and isolation width of 0.7 *m/z*). Singly and unassigned charge states were rejected. Dynamic exclusion was enabled with a repeat count of 2, an exclusion duration of 20 seconds, and an exclusion mass width of ±10 ppm.

### Mass spectrometry analysis.

Protein identification and quantification were done with Integrated Proteomics Pipeline (IP2, Integrated Proteomics Applications, Inc.) using ProLuCID/Sequest, DTASelect2 and Census (87–90). Identified proteins that were detected in only 1 replicate per sample were filtered out from dataset. Protein FC was calculated by dividing the average normalized M2 intensity by the average normalized M1 intensity for each protein.

### Quantification and statistics.

Unless otherwise specified, statistical analyses were performed on GraphPad Prism v.9.0.2. Statistical tests used 2-tailed 1-way ANOVA and post hoc tests for multiple comparisons, or Student’s *t* test or Wilcoxon’s test for comparison between 2 groups. In the box-and-whisker plots, the lower bound of the box indicates the first quartile while the upper bound of the box indicates the third quartile. The middle line represents the median value. The whiskers represent the minimum and maximum values. Error bars represent standard deviation. A *P* value of less than 0.05 was considered significant.

### Additional reagents.

A complete list of reagents and resources utilized in this study can be found in Supplemental Tables 3–6.

### Data availability.

All data reported in figures can be found in the Supporting Data Values file in the supplemental materials. This study did not generate new unique reagents or report original code. The raw and processed bulk RNA-seq data of invasive GSCs treated with M2 CM are deposited in the NCBI (GEO GSE251777). Supplemental Datasets 1–3 are available for download.

## Author contributions

EAA, SK, and MKA conceived the project. EAA wrote the manuscript, performed most of the experiments, and prepared the figures. DW contributed to the experimental work and manuscript preparation. ZAM, KH, and RCO contributed to the experimental work. EAA, DW, ZAM, KH, RCO, KKHY, MKA, and SK contributed to editing and revising the manuscript.

## Supplementary Material

Supplemental data

Supplemental data set 1

Supplemental data set 2

Supplemental data set 3

Supporting data values

## Version 1 08/22/2025

Electronic publication
