# Supplementary material for: An engineered glioblastoma model yields macrophage-secreted drivers of invasion
Source: JCI Insight. 2025 Aug 22;10(16):e181903. doi: 10.1172/jci.insight.181903 (PMC12406725; doi:10.1172/jci.insight.181903)
Supplement: Supplemental data [file jciinsight-10-181903-s040.pdf]

Title: An engineered glioblastoma model yields macrophage-secreted drivers of invasion

Authors: Erin A. Akins[1,2], Dana Wilkins[1,2], Zaki Abou-Mrad[3], Kelsey Hopland[3], Robert Osorio[4], Kenny Kwok Hei Yu[3], Manish K. Aghi[4], Sanjay Kumar[1,2,5,*]

Supplemental Figures

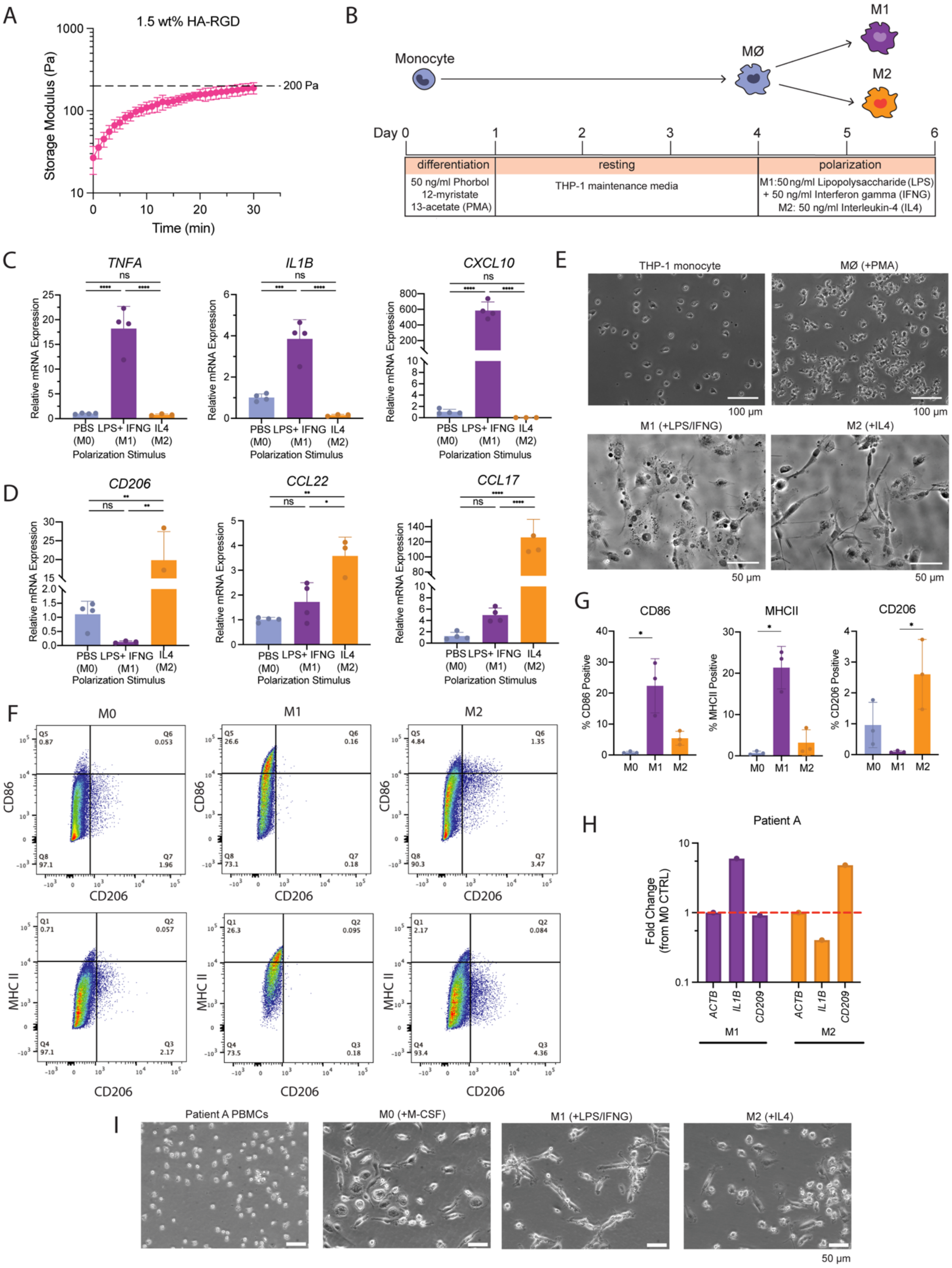

A
1.5 wt% HA-RGD
Storage Modulus (Pa)
200 Pa
Time (min)
B
Monocyte
MØ
M1
M2
Day 0
differentiation
resting
polarization
50 ng/ml Phorbol 12-myristate 13-acetate (PMA)
THP-1 maintenance media
M1:50 ng/ml Lipopolysaccharide (LPS) + 50 ng/ml Interferon gamma (IFNG)
M2: 50 ng/ml Interleukin-4 (IL4)
C
TNFA
IL1B
CXCL10
Relative mRNA Expression
PBS (M0)
LPS+ IFNG (M1)
IL4 (M2)
Polarization Stimulus
D
CD206
CCL22
CCL17
E
THP-1 monocyte
MØ (+PMA)
M1 (+LPS/IFNG)
M2 (+IL4)
100 µm
50 µm
F
M0
M1
M2
CD86
MHC II
CD206
G
CD86
MHCII
% CD86 Positive
% MHCII Positive
% CD206 Positive
H
Patient A
Fold Change (from M0 CTRL)
ACTB
IL1B
CD209
I
Patient A PBMCs
M0 (+M-CSF)
M1 (+LPS/IFNG)
M2 (+IL4)
50 µm

Supplemental Figure 1. Hydrogel characterization and THP-1 macrophage differentiation and polarization validation. **(A)** Hydrogel storage modulus (G') (in Pascals) versus time after the crosslinker is added. Time=0 represents the intersection of storage modulus (G') and loss modulus (G'') (n=3 hydrogels). **(B)** Schematic of THP-1 differentiation and polarization timeline. **(C and D)** qPCR gene expression of (**C**) M1 and (**D**) M2 markers in THP-1-derived macrophages polarized with LPS + IFNG or IL4 (n=3 biological replicates). **(E)** Representative phase images of THP-1 monocytes/macrophages throughout differentiation and polarization process. **(F and G)** Flow cytometry of M1 (CD86 and MHCII) and M2 (CD206) marker expression in THP-1 derived macrophages (n=3 biological replicates) (**F**) representative flow cytometry plots (**G**) quantification of % positive populations. **(H)** Representative qPCR gene expression of M1 and M2 markers in patient PBMC derived macrophages (n=5 patients). **(I)** Representative phase images of patient PBMC derived monocytes/macrophages throughout differentiation and polarization process (n=5 patients). Statistical significance was analyzed using one way ANOVA followed by Tukey's multiple comparison test (**C,D,E,G**). *P<0.05, **P<0.01, ***P<0.001, ****P<0.0001

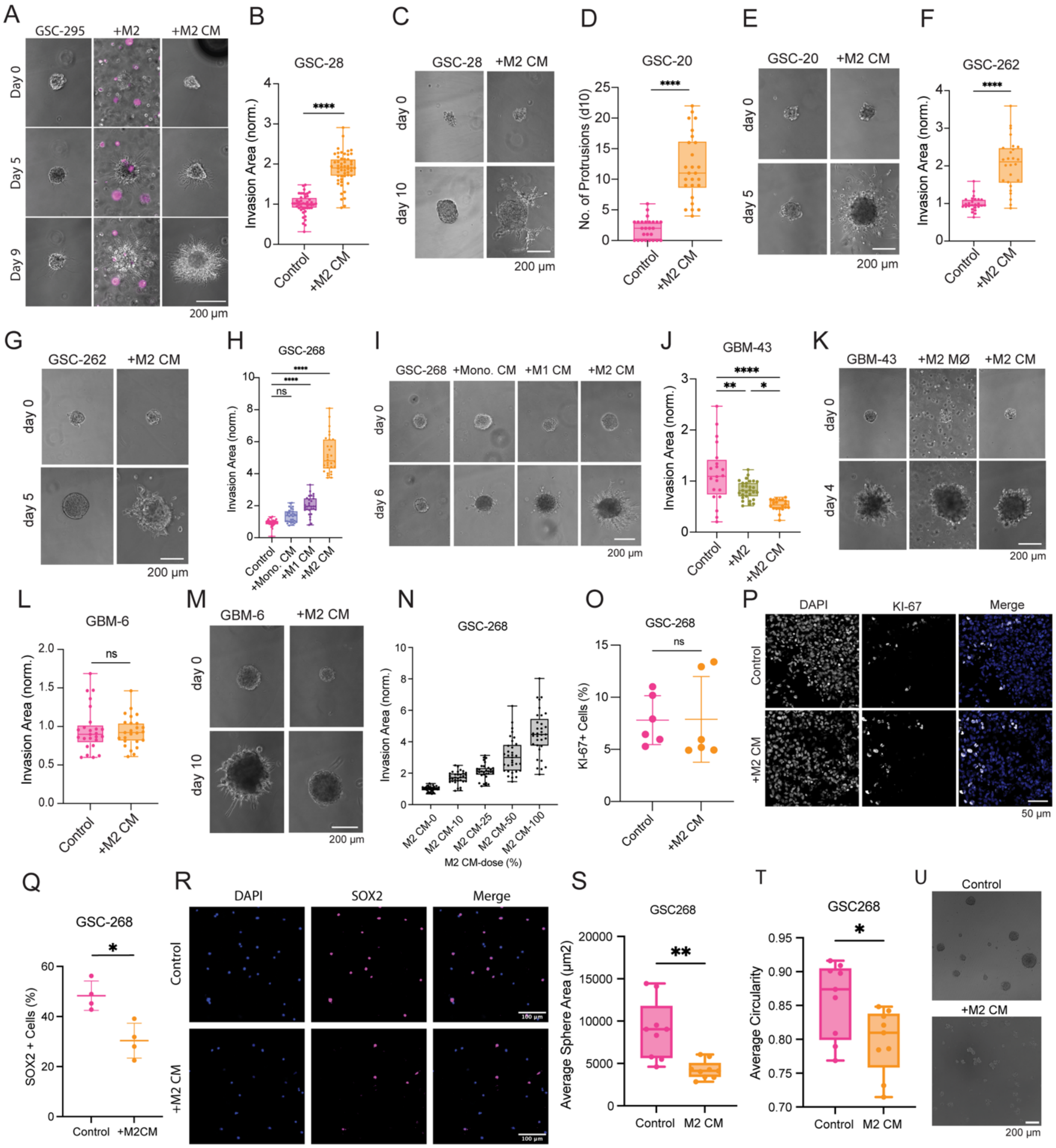

Supplemental Figure 2. Validation of M2 macrophage-induced invasion across multiple cell lines. (A) GSC-295 invasion assay with direct M2 macrophage co-culture and M2 macrophage conditioned media (CM). THP1s labeled in magenta. **(B** and **C)**

GSC-28 invasion assay with M2 CM (n = 47-53 spheres) (**B**) quantification and (**C**) representative phase images. **(D** and **E)** GSC-20 invasion assay with M2 CM (n=16 spheres) (**D**) quantification and (**E**) representative phase images. **(F** and **G)** GSC-262 invasion assay with M2 CM (n=26 spheres) (**F**) quantification and (**G**) representative phase images. **(H** and **I)** GSC-268 invasion assay with CM from monocytes (mono.), M1 macrophages, and M2 macrophages (n=30 spheres) (**H**) quantification and (**I**) representative phase images. **(J** and **K)** GBM-43 invasion assay with direct M2 macrophage co-culture and M2 CM (n = 20-29 spheres) (**J**) quantification and (**K**) representative phase images. **(L** and **M)** GBM-6 invasion assay with M2 CM (n=25 spheres) (**L**) quantification and (**M**) representative phase images. **(N)** GSC-268 invasion assay with varied doses of M2 CM quantification through serial dilutions of M2 CM (n=33 spheres). **(O** and **P)** KI-67 staining of fixed GSC-268 cells in 3D invasion assay (n=6 hydrogels) (**O**) quantification and (**P**) representative immunofluorescent images. **(Q** and **R)** Immunostaining for SOX2 expression in fixed GSC-268 encapsulated as single cells (n=4 hydrogels) (**Q**) quantification and (**R**) representative immunofluorescent images. **(S**-**U)** Sphere formation assay with GSC-268 cells (n=9 wells) (**S**) average sphere area (**T**) average sphere circularity (**U**) representative phase images. Spheroid invasion results were pooled across at least 2-3 independent replicates. Statistical significance was analyzed using an unpaired two-sided Student's t test (**B,D,F,L,O,G,S,T**) or a one way ANOVA followed by Tukey's multiple comparison test (**H,J**). *P<0.05, **P<0.01, ****P<0.0001

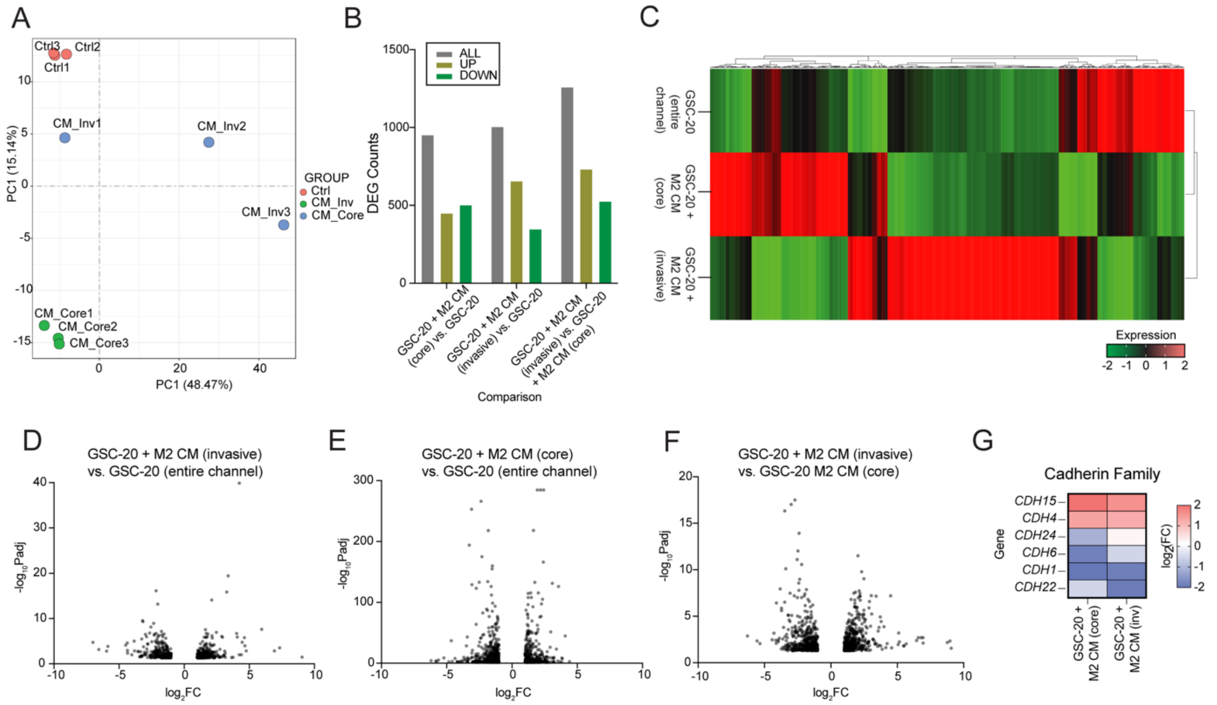

Supplemental Figure 3. Bulk RNA-seq quality control and additional analysis. (A) Principal Component Analysis (PCA) of bulk RNA-seq samples. **(B)** Bar graph showing up, down, and total differentially expressed gene (DEG) counts for each pairwise comparison using cut offs abs(log2FC) > 1 and P_{adj} < 0.05. **(C)** Heat map illustrating relative differential gene expression changes across samples. **(D-F)** Volcano plots of DEGs for (**D**) GSC-20 invasive fraction of M2 CM devices and entire channel of GSC-20 control devices, (**E**) GSC-20 core fraction of M2 CM devices and entire channel of GSC-20 control devices and (**F**) GSC-20 invasive fraction of M2 CM devices and GSC-20 core fraction of M2 CM devices. **(G)** Heat map showing GSC-20 relative gene expression levels of cadherin family genes obtained by bulk RNA-seq of cells isolated from invasion devices. Expression levels normalized to GSC-20 devices in control media (entire channel).

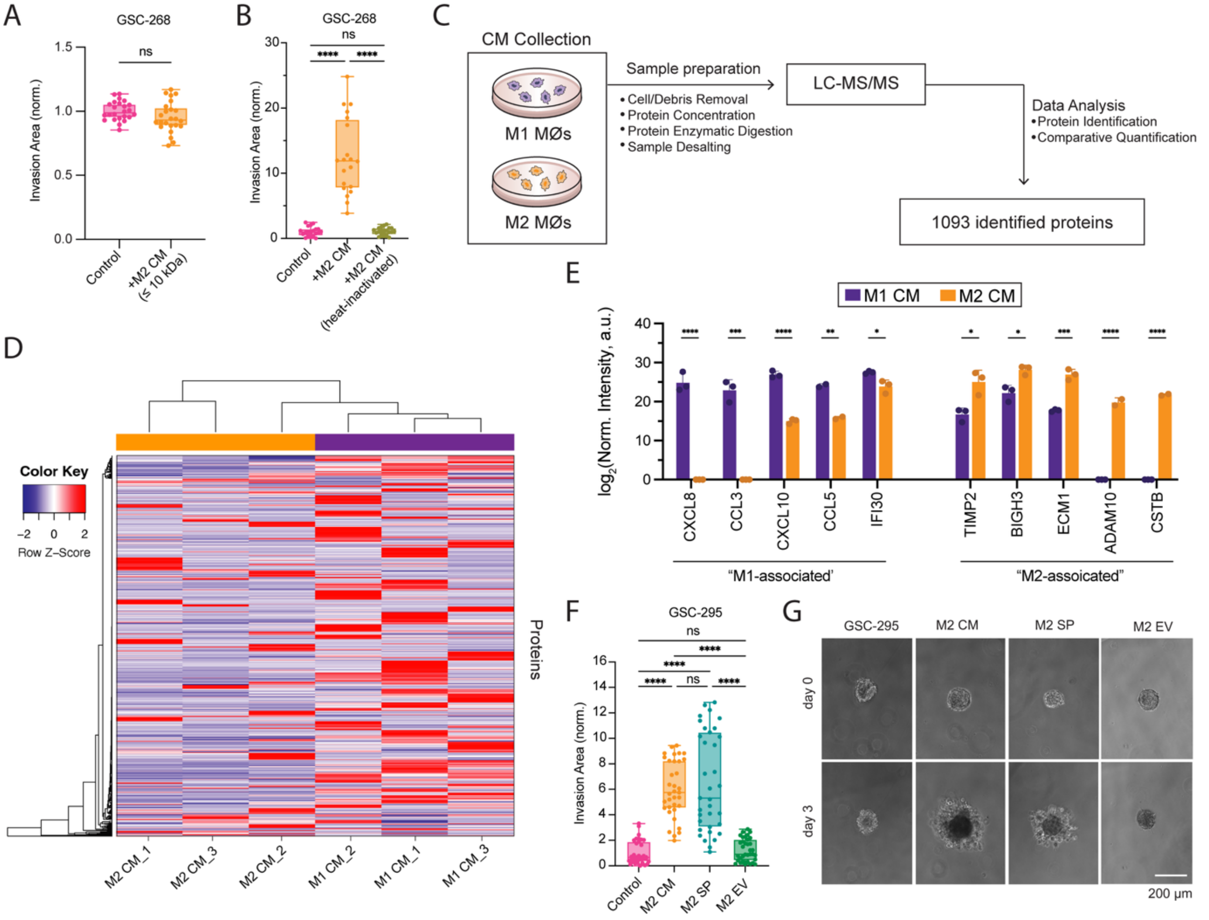

Supplemental Figure 4. Mass Spectrometry quality control and additional analysis. (A) GSC-268 invasion assay quantification using size-based filtered M2 CM (≤ 10 kDa fraction) (n=24 spheres). **(B)** GSC-268 invasion assay quantification using heat-inactivated M2 CM (n=20 spheres). **(C)** Schematic of mass spectrometry sample collection, preparation, and analysis. **(D)** Heat Map illustrating differential protein expression across samples. **(E)** Relative protein intensity of M1- and M2-associated proteins identified in M1 and M2 CM. **(F** and **G)** GSC-295 invasion assay with M2 CM, M2 soluble proteins (SP) and M2 extracellular vesicles (EV) (n= 35 spheres) (**F**) quantification and (**G**) representative phase images. Spheroid invasion results were pooled across at least 2-3 independent replicates. Statistical significance was analyzed

using unpaired two-sided Student’s t test (**A,E**) or a one-way ANOVA followed by Tukey’s multiple comparison test (**B,F**). * $P<0.05$, **$P<0.01$, ***$P<0.001$, ****$P<0.0001$

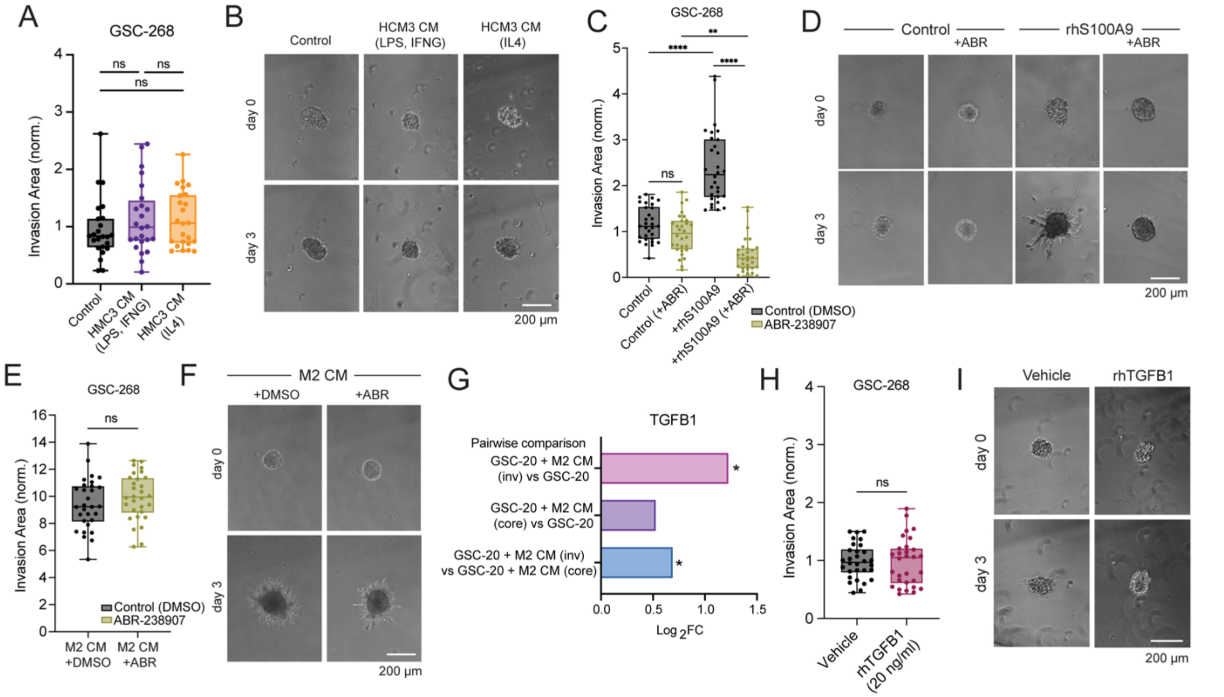

Supplemental Figure 5. Validation of TAM-derived BIGH3 specificity and intracellular pathway. (A and B) GSC-268 invasion assay with HMC3 CM (labeled with polarization stimuli) (n=24 spheres) (**A**) quantification and (**B**) representative phase images. **(C and D)** GSC-268 invasion assay with 5 μg/ml rhS100A9 and 25 μM ABR-238907 (ABR) (n=30 spheres) (**C**) quantification and (**D**) representative phase images. **(E and F)** GSC-268 invasion assay with M2 CM and 25 μM ABR-238907 (ABR) (n= 30 spheres) (**E**) quantification and (**F**) representative phase images. **(G)** Bar plot showing differential *TGFB1* gene expression across sample comparisons from bulk RNA-seq dataset. **(H and I)** GSC-268 invasion assay with 20 ng/ml rhTGFB1 (n=30 spheres) (**H**) quantification and (**I**) representative phase images. Spheroid invasion results were pooled across at least 2-3 independent replicates. Statistical significance was analyzed using a one-way ANOVA followed by Tukey's multiple comparison test (**A**) or Šídák's

multiple comparisons test (**C**) and unpaired two-sided Student's t test (**E,H**). *P<0.05, **P<0.01, ****P<0.0001

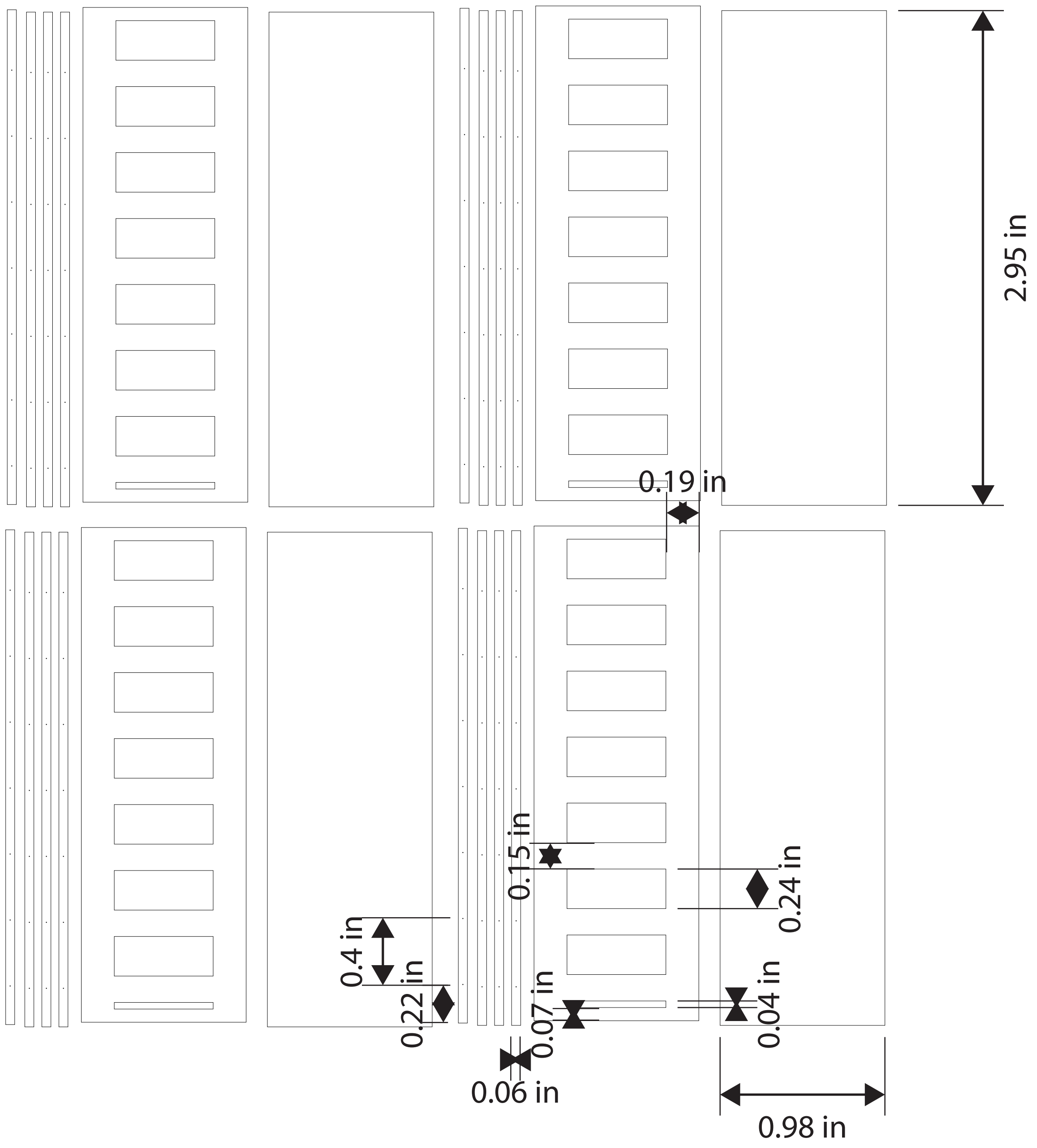

Supplemental Figure 6. Laser cutter designs for invasion device fabrication. Created using Adobe Illustrator.

Supplemental Table 1. GBM cell line sex, classification, and source.

Cell line	Patient Sex	Classification	Source	Reference
GSC-268	M	Classical	MD Anderson	#40
GSC-28	M	Mesenchymal	MD Anderson	#40
GSC-20	F	Mesenchymal	MD Anderson	#40
GSC-11	F	Proneural	MD Anderson	#40
GSC-262	M	Proneural	MD Anderson	#40
GSC-295	M	Proneural	MD Anderson	#40
GBM-43	M	Proneural	Mayo Clinic	#41
GBM-6	M	Classical	Mayo Clinic	#41
THP-1	M	N/A	ATCC	N/A
HMC3	Unspecified	N/A	ATCC	N/A
Patient A PBMCs	F	N/A	MSKCC	N/A
Patient B PBMCs	F	N/A	MSKCC	N/A
Patient C PBMCs	M	N/A	MSKCC	N/A
Patient D PBMCs	M	N/A	MSKCC	N/A
Patient E PBMCs	F	N/A	MSKCC	N/A

Supplemental Table 2. qPCR Primer Sequences

Target	Forward	Reverse
CXCL10	TGGCATTCAAGGAGTACCTCTC	TTCTTGATGGCCTTCGATTC
IL1B	TTCGACACATGGGATAACGAGG	TTTTTGCTGTGAGTCCCGGAG
TNFA	AACCTCCTCTCTGCCATCAA	CCAAAGTAGACCTGCCCAGA
CD206	GGGTTGCTATCACTCTCTATGC	TTTCTTGTCTGTTGCCGTAGTT
CCL22	ATCGCCTACAGACTGCACTC	GACGGTAACGGACGTAATCAC
CCL17	GAGCCATTCCCCTTAGAAAG	AGGCTTCAAGACCTCTCAAG
ACTB	ATTGCCGACAGGATGCAGAA	GCTGATCCACATCTGCTGGAA
BIGH3	CACTCTCAAACCTTTACGAGAC	CGTTGCTAGGGGCGAAGATG
GAPDH	GCATCCTGGGCTACACTGAG	GTCAAAGGTGGAGGAGTGGG
CD44	CGAACAAGGTGTGGGCAGAA	CCGTTGAGTCCACTTGGCTT
CEBPB	GGCCGGTTTCGAAGTTGATG	GACAGTTACACGTGGGTTGC
SERPINE1	TTTCAGAGGTGGAGAGAGCC	CTGATTTGTGGAAGAGGCGG
STAT3	ACCTGCAGCAATACCATTGAC	AAGGTGAGGGACTCAAACTGC
SNAI1	CACTATGCCGCGCTCTTTC	CTGCTGGAAGGTAAACTCTGGATTA
SNAI2	AACTGGACACACATACAGTGAT	GCGGTAGTCCACACAGTGAT
TWIST1	AGCTACGCCTTCTCGGTCT	TCTCTGGAAACAATGACATCTAGG
TGFB1	CACGTGGAGCTGTACCAGAA	TGCAGTGTGTTATCCCTGCT
ZEB1	TTACACCTTTGCATACAGAACC	TTTACGATTACACCCAGACTGC
OLIG2	TGGCTTCAAGTCATCCTCGTC	ATGGCGATGTTGAGGTCGTG
PDGFRA	TTGCGGAATAACATCGGAGGA	ATTAGGCTCAGCCCTGTGAGA
RSP9	GACTCCGGAACAAACGTGAGG	CTTCATCTTGCCCTCGTCCA
CD209	GCTGAGGAGCAGAACTTCCT	GTTGGGCTCTCCTCTGTTCC

Supplemental Table 3. Key Resources – Antibodies, Commercial Assays, Cell lines, & Virus Strains

Reagent / Resource	Source	Identifier
**Antibodies & Flow Reagents**		
Rabbit anti-BIGH3	Proteintech	10188-1-AP
Rabbit anti-S100A9	Abcam	ab63818
Rabbit IgG	Sigma-Aldrich	I5006
DAPI	Roche	10236276001
CellTracker	Thermo Scientific	C2925
Rabbit Anti-KI-67 [SP6]	Abcam	ab16667
Goat anti-Rabbit IgG (H+L) Highly Cross-Adsorbed Secondary Antibody, Alexa Fluor™ 647	Invitrogen	A-21245
Rabbit anti-SOX2	Invitrogen	PA1-094
Human TruStain FcX	Biolegend	422301
UltraComp eBeads Plus	Invitrogen	01-3333-41
PE/Dazzle 594 anti-human CD86	Biolegend	374217
BV421 anti-human CD206	Biolegend	321125
APCFire 750 anti-human HLA-DR	Biolegend	307657
Zombie Aqua Dye	Biolegend	77143
**Critical commercial assays**		
RNeasy Plus Micro Kit with gDNA eliminator columns	Qiagen	74034
iScript cDNA Synthesis Kit	BioRad	1708891
Applied Biosystems PowerUp SYBR Green Master Mix	Thermo Scientific	A25918
Aggrewell 400 24-well plates and Rinsing Solution	Stemcell Technologies	34415
**Experimental models: Cell lines**		
GBM-43	Mayo Clinic	N/A
GBM-6	Mayo Clinic	N/A
GSC-11	M.D. Anderson	N/A
GSC-268	M.D. Anderson	N/A
GSC-28	M.D. Anderson	N/A
GSC-262	M.D. Anderson	N/A
GSC-20	M.D. Anderson	N/A
GSC-295	M.D. Anderson	N/A
THP-1	ATTC	TIB-202
HMC3	ATCC	CRL-3304
Patient PBMCs	MSKCC	N/A
**Bacterial and virus strains**		
CAG-GFP lentivirus	Cellomics	PLV-10057-50

Supplemental Table 4. Key Resources - Chemicals, peptides, and recombinant proteins

Reagent / Resource	Source	Identifier
methacrylic anhydride (94%)	Sigma-Aldrich	760-93-0
sodium hyaluronate (Research Grade, 66 kDa – 99 kDa)	Lifecore Biomedical	HA60K-5
Integrin Binding Peptide	Anaspec	AS-62349
protease-cleavable peptide (KKCG-GPQGIWGQ-GCKK)	Genscript	N/A
Phorbol 12-myristate 13-acetate (PMA)	PeproTech	1652981
Interferon gamma (IFNG)	Bio Basic	RC217-17
Lipopolysaccharides (LPS)	Sigma-Aldrich	L2630-10MG
Interleukin-4 (IL4)	Bio Basic	RC212-15-5
Macrophage colony-stimulating factor (M-CSF)	Thermo Scientific	300-03-200UG
Penicillin-streptomycin	Gibco	15140122
HEPES (1M)	Gibco	15630080
2-Mercaptoethanol	Sigma-Aldrich	M3148-25ML
Amphotericin B solution	Sigma-Aldrich	A2942
Puromycin	Invitrogen	A1113803
Hyaluronidase from bovine testes, Type IV-S	Sigma-Aldrich	H3884
TRIzol Reagent	Invitrogen	15596018
Chloroform	Sigma	c2432
2-Propanol	Sigma-Aldrich	190764-4L
Glycogen, RNA grade	Thermo Scientific	R0551
Recombinant human BIGH3	R&D Systems	3409-BG
Recombinant human S100A9	R&D Systems	9254-S9
Recombinant human TGFB1	R&D Systems	240-B
Recombinant human EGF	R&D systems	236-EG
Recombinant human FGF	R&D systems	233-FB
Temsirolimus	MedChemExpress	HY-50910
ABR-238907	MedChemExpress	HY-141537
MHY1845	SelleckChem	S7811

Supplemental Table 5. Key Resources - Other

**Reagent / Resource**	**Source**	**Identifier**
RNA-sequencing services	Novogene Corporation Inc.	https://www.novogene.com/us-en/
Mass Spectrometry services	Vincent J. Coates Proteomics/Mass Spectrometry Laboratory at UC Berkeley	https://qb3.berkeley.edu/facility/pmsl/
DMEM/F12 50/50 1X	Corning	10-090-CV
25mm 0.45μm sterile cellulose acetate filter	VWR	76479-040
B-27 Supplement	Gibco	17504-044
Macrophage-SFM	Gibco	2065074
Fetal Bovine Serum (FBS)	Corning	MT 35-010-CV
phenol red-free serum-free Dulbecco's Modified Eagle's Medium (DMEM)	Thermo Fisher Scientific	21-063-029
Glutamax	Thermo Fisher Scientific	35-050-061
0.25% Trypsin-EDTA	Thermo Fisher Scientific	25200-072
Accutase cell detachment solution	Innovative Cell Technologies	490007-741
RPMI 1640 Medium	Gibco	11875093
MEM non-essential amino acids	Gibco	11140050
sodium pyruvate	Gibco	11360070
CLAREX Precision Thin Sheet, 1.5 mm	Astra Products	N/A
Glass microscope slide	Fisherbrand	12-550-A3
Cleaning Wires (0.00695 mm outer diameter)	Hamilton	18302

Supplemental Table 6. Deposited Data and Software and Algorithms

**Reagent / Resource**	**Source**	**Identifier**
**Deposited data**		
scRNA-seq GBM dataset	PMID: 34434898	GSE131928
scRNA-seq GBM TAMs dataset	PMID: 29262845	N/A
Receptor-Ligand dataset	PMID: 33147626<br>PMID: 26198319	N/A
RNA-seq dataset	This paper	GSE251777
scRNA-seq GBM for Cell Chat	PMID: 29091775	GSE84465
**Software and algorithms**		
Pathway Enrichment Analysis	Enrichr	https://maayanlab.cloud/Enrichr/
TCGA	GlioVis	http://gliovis.bioinfo.cnio.es/
Graphpad Prism 8.0	GraphPad Software	https://www.graphpad.com/
ImageJ	NIH	https://ImageJ.nih.gov/ij/
Adobe Illustrator	Adobe	https://www.adobe.com/
R 4.2.2	Comprehensive R Archive Network	https://cran.r-project.org
Seurat 5.0.3	Comprehensive R Archive Network	https://cran.r-project.org/web/packages/Seurat/index.html
CellChat 1.6.1	CellChat	http://www.cellchat.org

Dataset S1 (separate file). Differentially expressed genes identified by bulk RNA-seq.

Dataset S2 (separate file). Differentially expressed proteins identified by mass spectrometry analysis of M1 and M2-polarized macrophage conditioned media.

Dataset S3 (separate file). Receptor-ligand pairs identified from receptor-ligand analysis of GSC-20 transcriptome and THP-1 macrophage secreted proteins (proteomics).

Smalldevice_7unit_1channel (separate file). Adobe file with laser cutter designs for invasion devices.
